# Binding Modes of a Cytotoxic Dinuclear Copper(II) Complex with Phosphate Ligands Probed by Vibrational Photodissociation Ion Spectroscopy

**DOI:** 10.1021/acs.inorgchem.2c02091

**Published:** 2023-01-19

**Authors:** Marco Giampà, Davide Corinti, Alessandro Maccelli, Simonetta Fornarini, Giel Berden, Jos Oomens, Sabrina Schwarzbich, Thorsten Glaser, Maria Elisa Crestoni

**Affiliations:** † Department of Clinical and Molecular Medicine, Norwegian University of Science and Technology, Olav Kyrres Gate 9, 7030 Trondheim, Norway; ‡ Dipartimento di Chimica e Tecnologie del Farmaco, Università di Roma “La Sapienza”, I-00185 Roma, Italy; § Institute for Molecules and Materials, FELIX Laboratory, Radboud University, Toernooiveld 7, 6525 ED Nijmegen, The Netherlands; ∥ Lehrstuhl für Anorganische Chemie I, Fakultät für Chemie, Universität Bielefeld, D-33615 Bielefeld, Germany

## Abstract

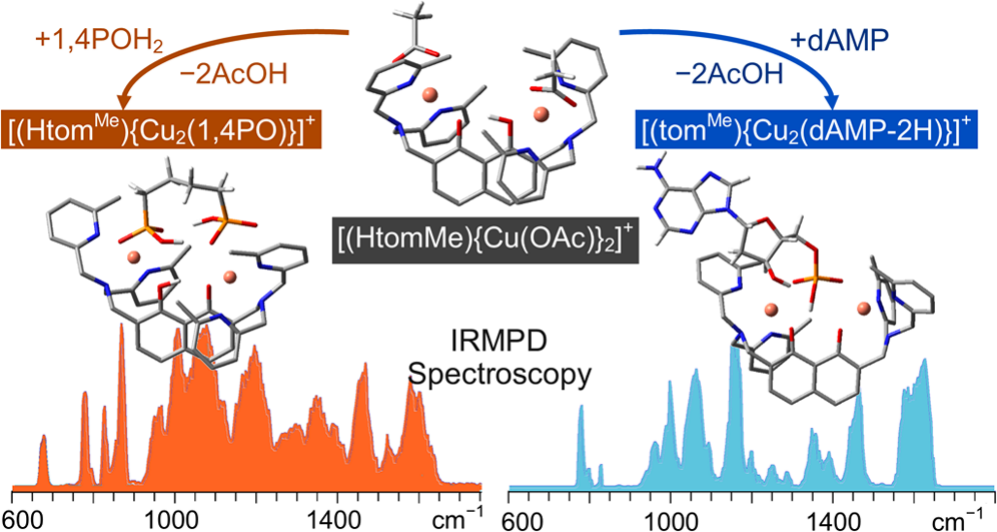

The dinuclear copper complex bearing a 2,7-disubstituted-1,8-naphthalenediol ligand, [(HtomMe){Cu(OAc)}_2_](OAc), a potential anticancer drug able to bind to two neighboring phosphates in the DNA backbone, is endowed with stronger cytotoxic effects and inhibition ability of DNA synthesis in human cancer cells as compared to cisplatin. In this study, the intrinsic binding ability of the charged complex [(HtomMe){Cu(OAc)}_2_]^+^ is investigated with representative phosphate diester ligands with growing chemical complexity, ranging from simple inorganic phosphate up to mononucleotides. An integrated method based on high-resolution mass spectrometry (MS), tandem MS, and infrared multiple photon dissociation (IRMPD) spectroscopy in the 600–1800 cm^–1^ spectral range, backed by quantum chemical calculations, has been used to characterize complexes formed in solution and delivered as bare species by electrospray ionization. The structural features revealed by IRMPD spectroscopy have been interpreted by comparison with linear IR spectra of the lowest-energy structures, revealing diagnostic signatures of binding modes of the dinuclear copper(II) complex with phosphate groups, whereas the possible competitive interaction with the nucleobase is silenced in the gas phase. This result points to the prevailing interaction of [(HtomMe){Cu(OAc)}_2_]^+^ with phosphate diesters and mononucleotides as a conceivable contribution to the observed anticancer activity.

## Abstract 2

Electrospray ionization mass spectrometry and IR ion spectroscopy allowed the vibrational and structural characterization of the adducts between a novel copper-containing antineoplastic drug candidate and several phosphate-containing ligands, including nucleotides. Comparison with DFT-calculated structures unambiguously showed a general preferential interaction of the complex with the phosphate moieties of the ligands, confirming the intended mechanism, which involves a strong binding between the drug and the phosphate groups in the DNA backbone.

## Introduction

The well-established use of metallodrugs as antimicrobial and anticancer agents introduces new schemes for medical treatment and holds promise for future improvements.^1^ Their multifaceted modes of action are mainly related to either substitution reactions at the metal center that leas to strong coordinative bindings to biological nucleophiles or redox activity that may trigger oxidative stress. Several factors, including the nature and possible oxidation states of the metal ion, its different ligands, and the geometry of the complex, need to be considered in tuning the metallodrug properties.^2^ Due to the complexity of these systems, a rational design is required to improve binding selectivity and spectrum of pharmacological activity.

A common mechanism of action of metallodrugs consists of the interaction with nucleic acids that produces changes in the three-dimensional (3D) structure, thus hampering genetic information transcription.^3^ Nucleobases are mostly involved in metal binding,^4^ and platinum-based chemotherapeutics, including cisplatin, preferentially bind to N7 of either guanine or adenine, resulting in 1,2-GG/AG intrastrand crosslinks and structural distortion of DNA helices.^5^ Recent contributions have accurately described these interactions also in the gas phase at the molecular level by infrared multiple photon dissociation (IRMPD) spectroscopy, thus unveiling binding motifs involving (thio)nucleobases and simple molecular targets.^6−10^

To limit side effects and elude chemoresistance of platinum-based drugs, new families of cytotoxic complexes based on different metals are currently under active investigation, including antiproliferative gold compounds,^11−13^ presenting innovative mechanisms of action and improved pharmacological features.

Many copper-based complexes^14^ have also been developed as effective antineoplastic agents, mainly through noncovalent contacts with DNA, which eventually cause a distortion of the nuclei.^15,16^ The modified response of cancer cells to copper is at the origin of several drug design strategies.^17^ Interestingly, the promising anticancer activity of a copper bis(thiosemicarbazone) derivative has prompted the radiolabeled preparation of the theranostic agent ^64^Cu(ATSM) conjugated to the tumor marker bombesin peptide for targeted delivery.^18^

**Figure 1:**
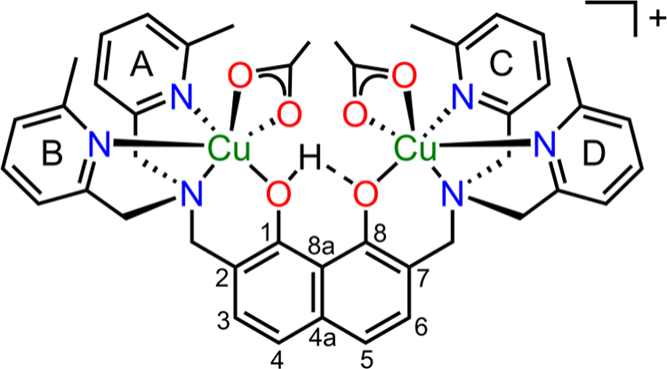
Dinuclear Cu_2_^II^ complex **[1(OAc)_2_]^+^**.

A recent alternative strategy intends to target the phosphate diesters of the DNA backbone as an alternative binding interaction. In a rational design—starting from the basic structure of copper complexes which mimic the action of endogenous hydrolytic enzymes such as nucleases and peptidases^19,20^ and whose reactivity entails the interaction of the metal with phosphate ester bonds during their catalytic cycles^21^—the dinuclear copper(II) complex [(Htom^Me^){Cu(OAc)}_2_]^+^, **[1(OAc)_2_]^+^** (Figure 1) has been developed.

The rigid 1,8-naphthalenediol ligand scaffold places the two copper ions at the same distance (6–7 Å) as two neighboring DNA phosphates. Two bulky bis-methylpyridine imino tridentate arms in the 2,7 positions sterically restrict binding with the less exposed nucleobases, thus favoring the coordination of the Cu^II^ ions to the oxygen atoms of two adjacent phosphates.^19^ The Cu^II^ ions in **[1(OAc)_2_]^+^** are coordinated pseudo-octahedrally, and the molecular structure exhibits a hydrogen atom bridge between two oxygen atoms of the 1,8-naphthalenediol ligand scaffold.

Based on biochemical ensemble methods and biophysical single-molecule methods, it was shown that **[1(OAc)_2_]^+^** exhibits only a low hydrolytic activity but binds irreversibly to the double-stranded DNA helix that induces intra- and interstrand interactions.^19^ A coherent model has been inferred that explains these observations: (i) **[1(OAc)_2_]^+^** binds two neighboring phosphate esters of the DNA, and (ii) the outwardly oriented and freely exposed naphthalene rings undergo intra- or interstrand π–π stacking interactions, which finally form aggregates.^20^

These interactions inhibit DNA synthesis in polymerase chain reaction (PCR) experiments and lead to selective, stronger cytotoxicity in cancer cells as compared to human stem cells, at a lower concentration in comparison with the anticancer drug cisplatin.^22^

Using a related Ni_2_^II^ complex where the four terminal 6-methylpyridine donors were exchanged with benzimidazole donors, the binding ability to two simple phosphate diester models, so far merely inferred in the case of **[1(OAc)_2_]^+^**, could be demonstrated.^23^ The strongly favored substitution of two coordinated acetate ligands by phosphates can occur in a bridging or in a terminal mode, as pointed out by following this process by ^1^H NMR spectroscopy.^24^ However, all of these evidences do not finally prove the binding of **[1(OAc)_2_]^+^** or the related Ni_2_^II^ complexes to the phosphates and not to the nucleobases of DNA.

Among the various analytical techniques lately employed to explore the reactivity of metal complexes with different biomolecules, electrospray ionization mass spectrometry (ESI-MS) has proven to be a powerful tool to examine the interactions of metal complexes with selected ligands at the molecular level.^25^ ESI-MS studies in medicinal inorganic chemistry may provide useful clues for characterizing the intrinsic features and unique mechanisms of metal-based agents, possibly proposing novel paths for the design of new drugs.

This contribution chiefly relies on ESI-MS to provide evidence for the selective binding of **[1(OAc)_2_]^+^** with representative phosphate diesters with varying chemical complexity, from a simple phosphate ion to an intact nucleotide isolated in a solvent-free environment. Possessing two possible P–O donors, each phosphate ligand can in principle support bidentate coordination. This binding mode is also favored in the absence of solvent molecules. Conversely, a monodentate interaction is exhibited in a dinuclear Ni^II^ complex with phosphate diester, assisted by hydrogen bonds of a coordinated water ligand.^24^

To improve our understanding of the charged species of interest, Fourier transform ion cyclotron resonance (FT-ICR) mass spectrometry^26^ and infrared multiple photon dissociation (IRMPD) spectroscopy^27−31^ have been employed in combination with energy-resolved collision-induced dissociation (CID) experiments and with density functional theory (DFT) calculations. IRMPD spectroscopy has been extensively exploited for the study of metal^8,32−36^ and halide^37,38^ ion binding patterns, isomeric discrimination,^39−42^ and identification of metabolites.^43−45^ The present analysis in the gas phase is aimed to reinforce the hypothesis of the primary interaction of the Cu_2_^II^ complex to phosphate groups as a leading driving force to its irreversible structural effect on DNA.

## Experimental Section

### Materials

All reagents and solvents used in this work were research grade products obtained from commercial sources (Merck-Sigma-Aldrich S.r.l., Milan, Italy) and used as received. The dinuclear copper complex [(Htom^Me^){Cu(OAc)}_2_](Oac), bis(acetate)-(μ-2,7-bis({bis[(6-methylpyridin-2-yl)methyl]amino}methyl)naphthalene-1,8-diolato)-di-copper(II), **[1(OAc)_2_]**(Oac), was synthesized, purified, and characterized, as described in the literature.^19^ A stock acetonitrile solution of **[1(OAc)_2_]^+^**, (C_44_H_47_Cu_2_N_6_O_6_), and methanol solutions of orthophosphoric acid (H_3_PO_4_); 1,2-ethylenediphosphonic acid (C_2_H_8_O_6_P_2_), 1,2-POH_2_; 1,4-butanediphosphonic acid (C_4_H_12_O_6_P_2_), 1,4-POH_2_; deoxyadenosine monophosphate (C_10_H_14_N_5_O_6_P), dAMP, and deoxyguanosine monophosphate (C_10_H_14_N_5_O_7_P), dGMP were prepared each in the millimolar range. The stock solution of **[1(OAc)_2_]^+^** was mixed with each of the methanolic solutions of the model ligands at room temperature in a 1:3 molar ratio. All of the so obtained solutions were diluted in acetonitrile to a final concentration of (1–4) × 10^–5^ M for **[1(OAc)_2_]^+^**. Each reaction mixture was submitted to electrospray ionization (ESI) by direct infusion using a syringe pump at a flow rate of 120 μL h^–1^.

### Mass Spectrometric Experiments

Electrospray mass spectra and energy-variable CID experiments were conducted using a commercial Paul-type ion trap (Esquire 3000+, Bruker Daltonics) and a hybrid triple quadrupole linear ion trap instrument (Applied Biosystem API 2000 Q-Trap) mass spectrometer with a Q1q2Q_LIT_ configuration (Q1, first mass analyzing quadrupole; q2, nitrogen filled collision cell; and Q_LIT_, linear ion trap). The precursor ions were mass-isolated and further submitted to dissociation by increasing values of collision energy (CE). Nitrogen and helium were used as collision gases in the 2000 Q-TRAP and Esquire 3000+ instruments, respectively. In the energy-resolved CID assays, the ions of interest were desolvated in the first region (Q0), mass-selected in Q1, and then allowed to collide with N_2_ at a nominal pressure of 1.9 × 10^–5^ mbar in the collision cell q2 at variable collision energies (*E*_lab_= 0–120 eV), thus inducing fragmentation. The dissociation pattern was monitored by scanning Q_LIT_ in the enhanced mode of operation, thus increasing both resolution and signal intensity. The relative ion abundances are corrected for the occurrence of a small amount of early dissociation products formed in the region before q2. Although quantitative threshold data are not directly accessible,^46^ a phenomenological threshold energy (TE) for the different fragmentation routes can be obtained from linear extrapolation of the rise of the breakdown curves attained by converting the collision energies to the center-of-mass frame (*E*_CM_ = [*m*/(*m* + *M*)]*E*_lab_, where *m* and *M* are the masses of the collision gas and of the precursor ion, respectively).^47−50^ Corrections for the nominal zero collision energy value were derived from retarding potential analyses (Figure S1). CID experiments were also performed in the Paul ion trap through radiofrequency excitation (40 ms) with an activation amplitude of 0.20–0.80 V, followed by collisions with the helium buffer gas. MS^3^ experiments were performed on peaks arising from the first dissociation step to verify each fragmentation route.

High-resolution mass spectrometry experiments were performed using a Bruker BioApex Fourier transform ion cyclotron resonance (FT-ICR) mass spectrometer (Bruker Daltonics GmbH, Bremen, Germany) equipped with an Apollo I ESI source operated in positive polarity mode, a 4.7 T superconducting magnet, and an infinity cell. The raw data were obtained by the Xmass software package and treated using the DataAnalysis program (Bruker Daltonics). These measurements allowed us to make confident assignments of the ions of interest.

Electrosprayed ions were accumulated in a radiofrequency-only hexapole ion guide for 1 s. After desolvation by a heated (400 K) N_2_ counter-flow drying gas, the ion population was driven into the infinity cell at 300 K and accumulated in a radiofrequency (rf)-only hexapole ion guide for 1 s. All mass spectra were acquired over a mass range of 100–1500 Da. The FT-ICR mass spectra were internally frequency-to-*m*/*z*-calibrated using ions of known elemental composition. All mass measurements are based on the “monoisotopic” ion. For high-resolution FT-ICR MS analyses, 100 scans were coadded with an acquisition size of 1 mega words.^51^

### IRMPD Spectroscopy

Infrared multiple photon dissociation (IRMPD) spectra were recorded at the Free Electron Laser for Infrared eXperiments (FELIX) facility (Nijmegen, The Netherlands)^52^ using a commercial 3D quadrupole ion trap mass spectrometer modified to permit optical access to the trapped ions (Bruker amaZon speed ETD).^53^ The charged complexes of interest were mass-selected in the ion trap and irradiated by a single IR pulse from the FEL to produce wavelength-dependent infrared multiple photon dissociation. FELIX was operated at 10 Hz with an energy of 70–100 mJ per pulse in the frequency range of 600–1800 cm^–1^ with steps of 3 cm^–1^. For each IR step, six replicate mass spectra were averaged. To prevent excessive depletion of the parent ions (saturation) and minimize the formation of fragment ions below the low mass cutoff of the MS, the spectra were recorded at several levels of laser pulse energy attenuation.^54^ IRMPD spectra were collected by plotting the photofragmentation yield *R* (*R* = −ln[*I*_P_/(*I*_P_ + ∑*I*_F_)]), where *I*_P_ and *I*_F_ are the abundances of the precursor ion and of a fragment ion, respectively, as a function of the wavenumber.^55^ The yield was linearly corrected for the frequency-dependent variation in laser pulse energy.^56^

### Computational Details

Density functional theory (DFT) calculations were carried out using Gaussian 09 rev.D01. The initial structure of **[1(OAc)_2_]^+^** was designed starting from the molecular structure previously reported.^19,20^ Guess structures of the dinuclear copper complexes with phosphate ligands L (L = H_3_PO_4_, 1,2-POH_2_, 1,4-POH_2_) were prepared by replacing the two acetate ligands with the doubly deprotonated ligand ions, eventually considering different possible binding motifs. The triplet electronic state was considered for all of the calculated species. In fact, singlet state calculations were preliminarily employed for the lowest-energy structures of the different complexes and found to be higher in energy than the corresponding open-shell structures by ca. 80 kJ mol^–1^, in agreement with the weak antiferromagnetic coupling already reported to be present in dinuclear Cu^II^ complexes with bridging diphosphonate ligands.^57^ All of the considered structures were submitted to optimization and frequency calculations at the B3LYP level using a double zeta quality basis set due to the high computational cost needed for open-shell species, in particular, 6-31+G(d) for O, N, and P atoms; 6-31G(d) for H and C; and the LanL2DZ effective-core potential for Cu. Harmonic frequencies were scaled by 0.97 on the basis of the good agreement thus obtained with IRMPD spectra. However, for vibrational modes involving PO bonds, typical scaling factors were found to be systematically too low; therefore, no scaling factor was used to treat and plot PO stretches of any kind following a procedure widely described in the literature.^58−63^ Calculated linear IR spectra have been convoluted with a Lorentzian profile of 12 cm^–1^ (full width at half-maximum (fwhm)). In addition, single-point energy calculations at the B3LYP-D3/def2TZVP and M06-2X-D3/def2TZVP level were performed on the B3LYP-optimized structures to compare the relative energies obtained by B3LYP with the ones computed by adding dispersion correction and using a functional with a higher percentage of HF exchange, respectively. To obtain relative enthalpies and Gibbs free energies at both B3LYP-D3 and M06-2X-D3 levels, B3LYP thermodynamic corrections were used.

## Results and Discussion

### Photodissociation and CID Mass Spectra

Exploring the stability and binding preferences of potential metallodrugs through the reaction with ligands representing recognized targets is valuable for their evaluation and development. In this study, ESI-MS was used to scrutinize the molecular composition, stoichiometries, and binding mode of metallodrug adducts^24,64^ to characterize and compare the interaction of the Cu_2_^II^ complex **[1(OAc)_2_]^+^** with exemplary phosphate-containing ligands, including nucleotides. The preferred binding of this Cu_2_^II^ complex to DNA phosphate diesters was already shown to be at the origin of its strong cytotoxic activity for cancer cells.^23,24^

After incubation with the selected compound L = AcOH, H_3_PO_4_, 1,2-POH_2_, 1,4-POH_2_, dAMP, dGMP, **[1(L-2H)]^+^** adducts were formed through the formal release of two acetate ligands. The adducts were observed after 5–10 min and persisted over 48 h. Lists of experimental *m*/*z* values and theoretical mass peaks ascribed to each dicopper species assigned by FT-ICR MS are reported in Table S1 with a confidence level <1 ppm.

When an acetonitrile solution of **[1(OAc)_2_]^+^** acetate is electrosprayed in the positive ion mode, a prominent peak corresponding to **[1(OAc)_2_]^+^** is observed, whose coordinated acetate ligands appear inert to substitution by the solvent.^22^ The high-resolution mass spectrum of **[1(OAc)_2_]^+^** presented in Figure S2 is consistent with [C_44_H_47_N_6_O_6_Cu_2_]^+^ composition and displays the distinct isotope pattern of a dinuclear copper complex.

While the high resolving power and mass accuracy attainable with FT-ICR MS^65^ are a valuable tool to monitor and reliably identify reaction products formed in solution, ESI-MS using a triple quadrupole or ion trap analyzer provides MS*^n^* options for structural characterization.^66^

CID experiments performed in a hybrid triple quadrupole linear ion trap show that the fragmentation of **[1(OAc)_2_]^+^** ions (*m*/*z* 881) occurs by sequential losses of two acetic acid molecules, leading first to ions at *m*/*z* 821 and then to *m*/*z* 761, releasing two vacant binding sites on each copper ion. At higher collision energies, as verified by MS^3^ experiments, competitive channels emerge that involve the tom^Me^ skeleton, namely, the cleavage of a bulky bis-methylpyridine imino tridentate arm [C_14_H_15_N_3_] and its copper complex [C_14_H_14_N_3_Cu]. From this set of experiments, a dissociation pattern could be established (Figure S3).

In the plot of energy-dependent CID of **[1(OAc)_2_]^+^** ions, the abundances of all secondary fragments are grouped together with the primary fragment ion at *m*/*z* 821, and the linear extrapolation of the rise of the sigmoid curve yields the appearance phenomenological threshold energy (TE) of 0.44 ± 0.20 eV, as shown in Figure S4. This extrapolation by no means affords a measure of the threshold energy for dissociation, yielding, however, a benchmark value taken as a basis for a comparative evaluation of fragmentation pathways undergone by dinuclear Cu_2_^II^ adducts with various phosphate ligands further investigated in this study (Table S2).

A similar fragmentation pattern confirming the elimination of acetic acid is observed when mass-selected **[1(OAc)_2_]^+^** is probed by IRMPD spectroscopy to gather structural information in the fingerprint range of the IR spectrum. As an example, the mass spectrum collected after photofragmentation of **[1(OAc)_2_]^+^** exposed to IR radiation at 1600 cm^–1^ is shown in Figure S5.

### Binding with Phosphate Ligands

The Cu_2_^II^ complex was allowed to react with model phosphate ligands aiming to elucidate the preferred binding mode of this potential anticancer metallodrug. When incubated with phosphoric acid, the prototypical phosphate ligand, **[1(OAc)_2_]^+^**, provides predominantly a singly charged ion at *m*/*z* 859, **[1(HPO_4_)]^+^**, corresponding to [C_40_H_42_N_6_O_6_PCu_2_]^+^ composition, where both acetate ligands are replaced, and a doubly charged species at *m*/*z* 430, **[1(H_2_PO_4_)]^2+^**, corresponding to [(C_40_H_42_N_6_O_6_PCu_2_)_2_H]^2+^ (Figure S6).

At variance with **[1(OAc)_2_]^+^**, the CID assay performed on the phosphoric acid adduct **[1(HPO_4_)]^+^** reveals fragmentation along two primary dissociation channels, namely, to ions at *m*/*z* 632, by loss of one bulky bis-methylpyridine amino tridentate arm [C_14_H_17_N_3_], and at *m*/*z* 761, by the formal release of the ligand, H_3_PO_4_.

**Figure 2:**
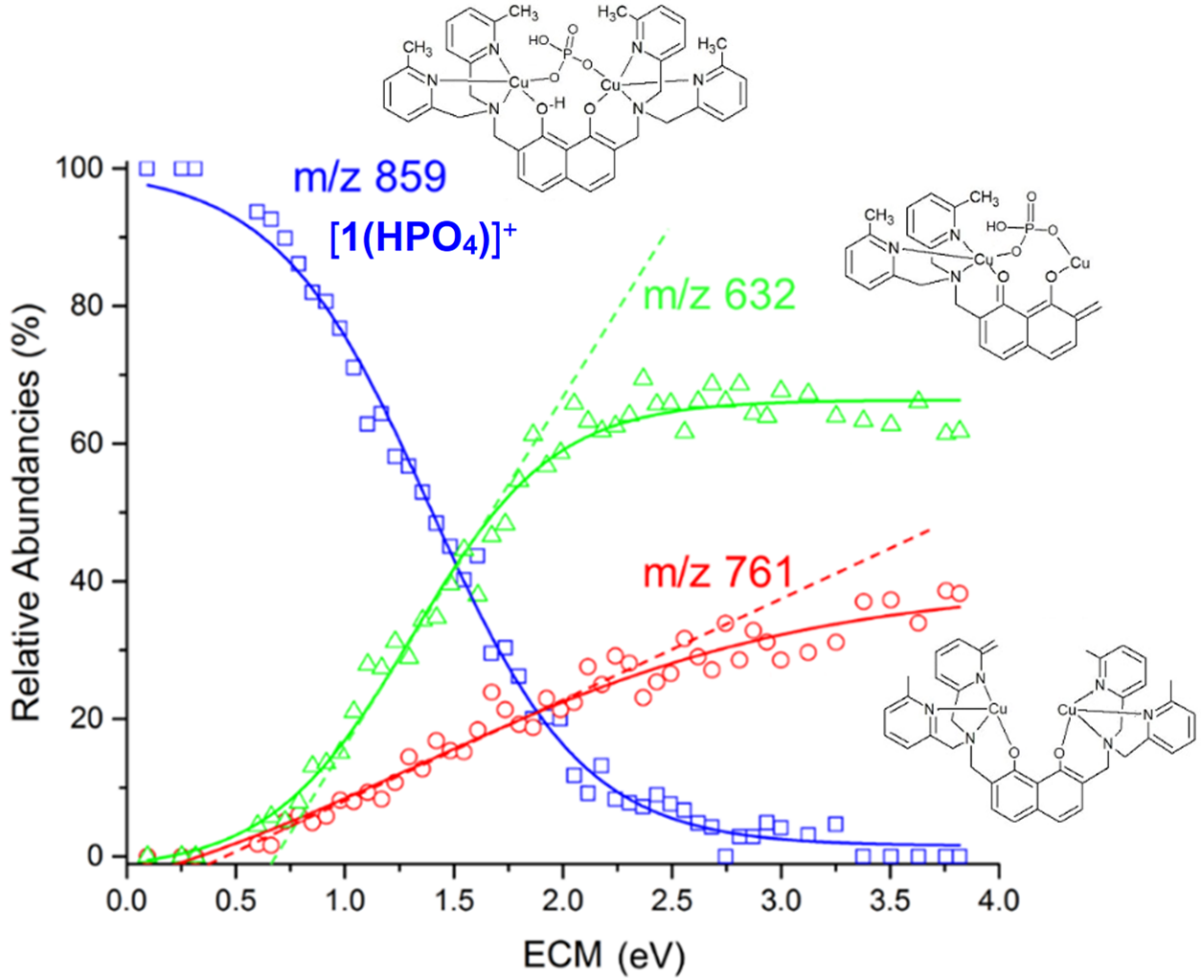
Relative abundances of mass-selected **[1(HPO_4_)]^+^** ions (blue profile, *m*/*z* 859) and product ions (red profile, *m*/*z* 761; green profile, *m*/*z* 632) as a function of collision energy (center of mass) during an energy-resolved CID experiment. Putative structure of precursor and fragment ions is reported.

Figure S7 shows the MS/MS spectrum and the assessed dissociation pattern validated by MS*^n^* experiments. Interestingly, the fragment ion *m*/*z* 632 mainly undergoes loss of the naphthalene unit, yielding a dicopper species (*m*/*z* 448) that still retains the phosphoric acid ligand, suggesting a significant affinity of this unit for the metal. The energy-resolved CID of **[1(HPO_4_)]^+^** displays the abundances of primary fragment ions at *m*/*z* 632 and 761 clustered together with their secondary dissociation products (Figure 2).

The extrapolated phenomenological TEs of 0.42 ± 0.2 and 0.70 ± 0.2 eV for the appearance of ions at *m*/*z* 761 and 632, respectively, indicate comparable importance of the two fragmentation paths at low collision energy (Table S2). However, the steeper raise of fragment *m*/*z* 632 as compared to *m*/*z* 761 suggests that the release of phosphoric acid from the complex follows a path presenting a tighter transition state,^67^ probably due to the occurrence of an isomerization reaction.

Further evidence of a direct, favorable Cu-phosphate contact has been gathered when two disphosphonate ligands with different chain lengths, i.e., 1,2-POH_2_ and 1,4-POH_2_, have been allowed to react with **[1(OAc)_2_]^+^**, giving rise to singly charged substitution products of both acetate ligands by the bifunctional molecules at *m*/*z* 951, **[1(1,2PO)]^+^**, corresponding to [C_42_H_47_N_6_O_8_P_2_Cu_2_]^+^ composition, and *m*/*z* 979, **[1(1,4PO)]^+^**, corresponding to [C_44_H_51_N_6_O_8_P_2_Cu_2_]^+^ (Figure S8). The activation of both **[1(1,2PO)]^+^** and **[1(1,4PO)]^+^** by energy-resolved CID proceeds through the exclusive loss of the amino tridentate arm [C_14_H_17_N_3_], yielding fragment ions *m*/*z* 724 and 752, characterized by comparable TE values of 0.53 ± 0.2 and 0.55 ± 0.2 eV, respectively (Figure S9 and Table S2). Conversely, no product ion derived from a direct elimination of the diphosphonate ligands is observed, at variance with the behavior observed in the presence of the acetate and phosphate anions.

Notably, the primary fragment of **[1(1,2PO)]^+^** at *m*/*z* 724 further dissociates by loss of copper-containing fragments [C_2_H_7_P_2_O_6_Cu] and [C_2_H_6_P_2_O_6_Cu_2_], suggesting 1,2POH to be directly involved in metal binding (Figure S10). In contrast, no subsequent dissociation steps take place in the case of **[1(1,4PO)]^+^** (Figure S11). This finding is consistent with a higher affinity of 1,4-butanediphosphonic acid for the sampled dinuclear copper complex and suggests that a four-carbon chain best accommodates the two phosphate binding sites on the metal ions.

### Binding with Nucleotides

To address the increasing ligand complexity, **[1(OAc)_2_]^+^** was exposed to intact purine nucleotides, either deoxyadenosine monophosphate (dAMP) or deoxyguanosine monophosphate (dGMP). Chosen as exemplary, simplified models of DNA components, they offer several potential competing binding sites, including phosphate and nucleobase groups. ESI-MS analysis provides singly charged products detected at *m*/*z* 1092, **[1(dAMP-2H)]^+^**, and *m*/*z* 1108, **[1(dGMP-2H)]^+^**, identified as [C_50_H_53_N_11_O_8_PCu_2_]^+^ and [C_50_H_53_N_11_O_9_PCu_2_]^+^, respectively (Figure S12). Doubly charged adducts at *m*/*z* 546.5, **[1(dAMPH-H)]^2+^** and *m*/*z* 554.5, **[1(dGMPH-H)]^2+^**, were also formed to a significant extent (Figures S12 and S13). CID mass spectra were obtained using an ion trap instrument, and the resulting CID mass spectra are summarized in Figure S14.

Experiments at variable CE conducted on both dinucleotide complexes, **[1(dAMP-2H)]^+^** and **[1(dGMP-2H)]^+^**, activate a major dissociation channel releasing the amino tridentate arm [C_14_H_17_N_3_], at the comparable TE value of 0.83 ± 0.2 eV. Such fragmentation behavior conforms to the one observed above for the two diphosphonate ligands, where the prominent skeletal fragmentation of the ligand is indicative of preferential binding of Cu ions for the phosphate-containing ligand (Figure S15). Also, the value is close to the TE for the same dissociation out of **[1(HPO_4_)]^+^**, suggesting that the three species share similar binding motifs. The lack of fragment ions involving loss of characteristic neutrals such as phosphoric acid or deoxyribosiumphosphate that might be diagnostic of metal–nucleobase coordination supports dicopper complex binding to the phosphate diester functionality to be the major binding motif. Parallel and consecutive paths confirmed by MS*^n^* experiments in the breakdown schemes allow the dissociation pattern of **[1(dAMP-2H)]^+^** and **[1(dGMP-2H)]^+^** to be defined (Figures S16 and S17, respectively).

### IRMPD Spectroscopy and Structural Assignment

**Figure 3:**
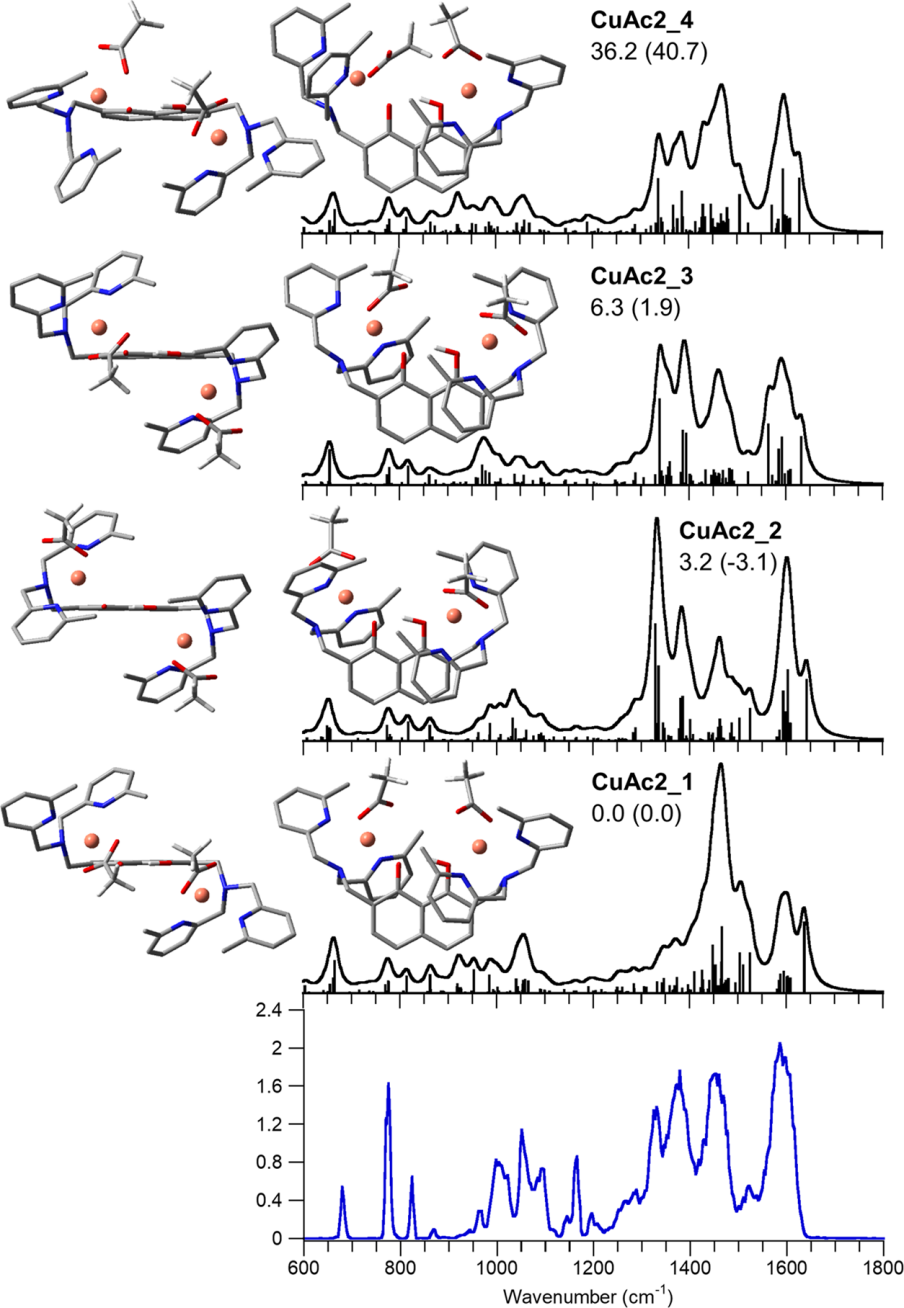
IRMPD spectrum of **[1(OAc)_2_]^+^** (bottom panel) compared with calculated IR spectra of selected conformers, whose optimized structures are reported, each from two different perspectives. Relative free energies (enthalpies in parentheses) at 298 K are in kJ mol^–1^.

**Figure 4:**
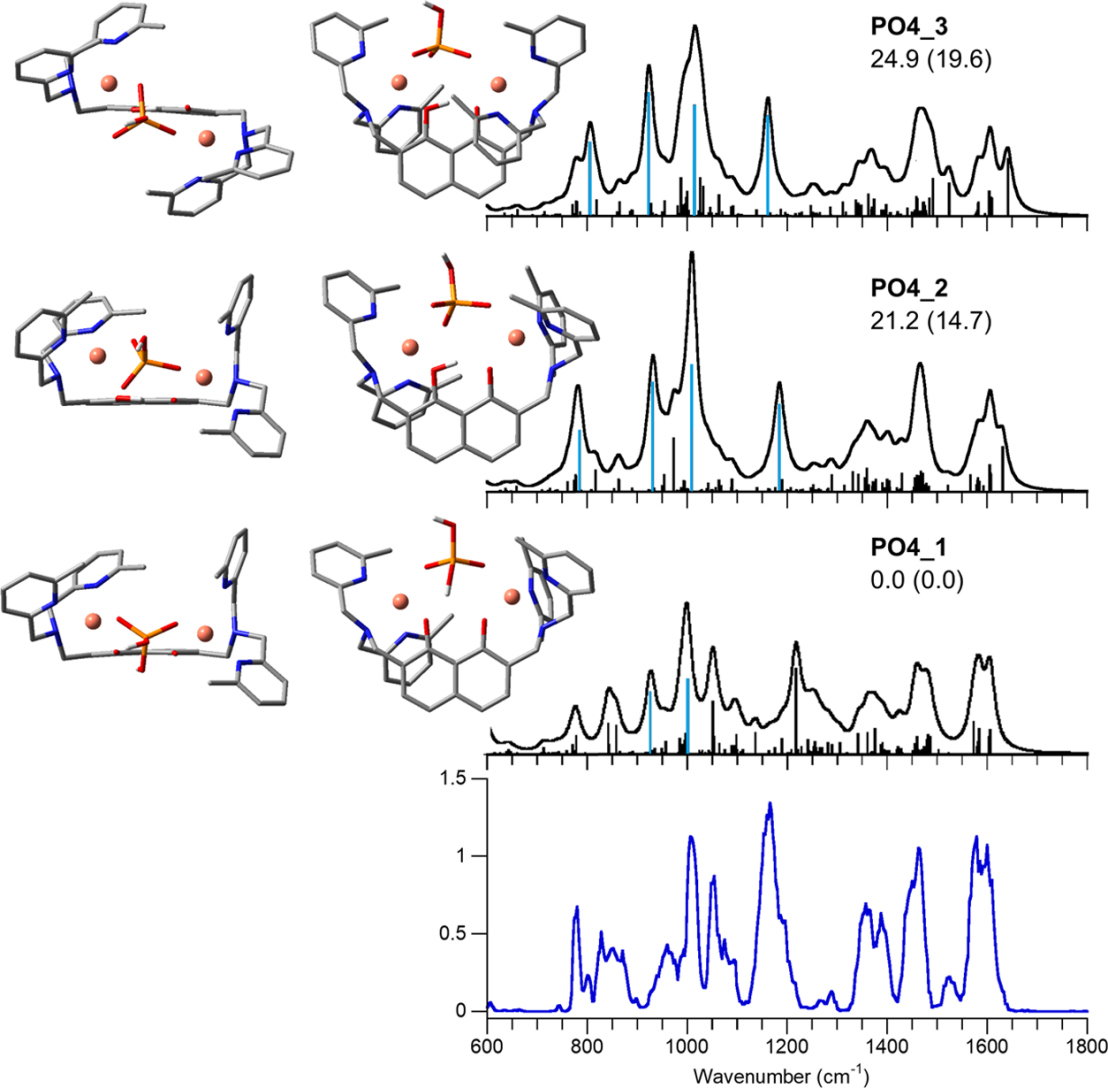
IRMPD spectrum of **[1(HPO_4_)]^+^** (bottom panel) compared with calculated IR spectra of selected conformers, whose optimized structures are reported each from two different perspectives. Relative free energies (enthalpies in parentheses) at 298 K in kJ mol^–1^. Unscaled vibrational modes are highlighted in pale blue.

**Figure 5:**
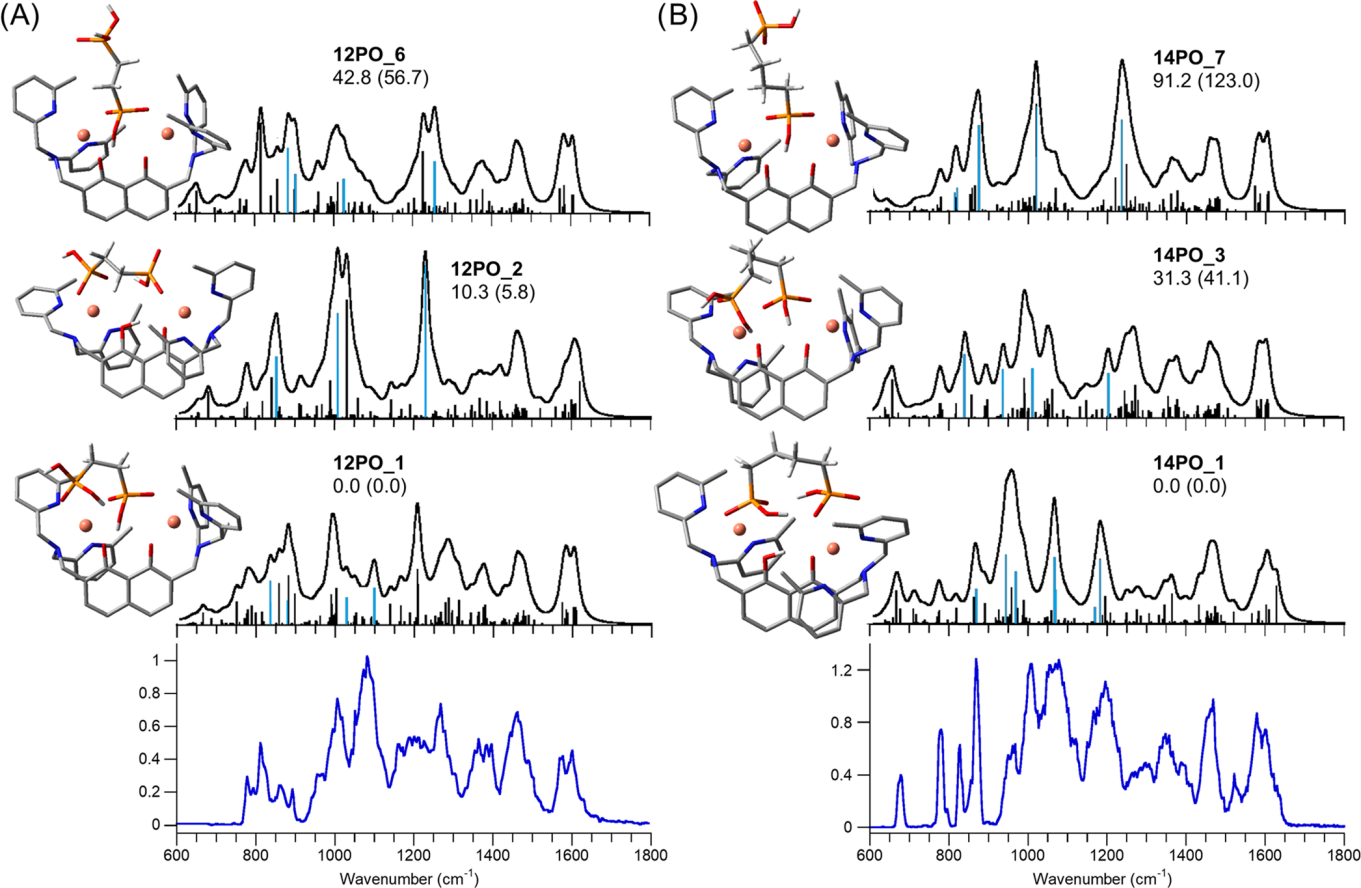
IRMPD spectra of **[1(1,2PO)]^+^** (panel (A), bottom) and **[1(1,4PO)]^+^** (panel (B), bottom) compared with calculated IR spectra of selected conformers, whose optimized structures are reported on the left of each spectrum. Relative free energies (enthalpies in parentheses) at 298 K are in kJ mol^–1^. Unscaled vibrational modes are highlighted in pale blue.

**Figure 6:**
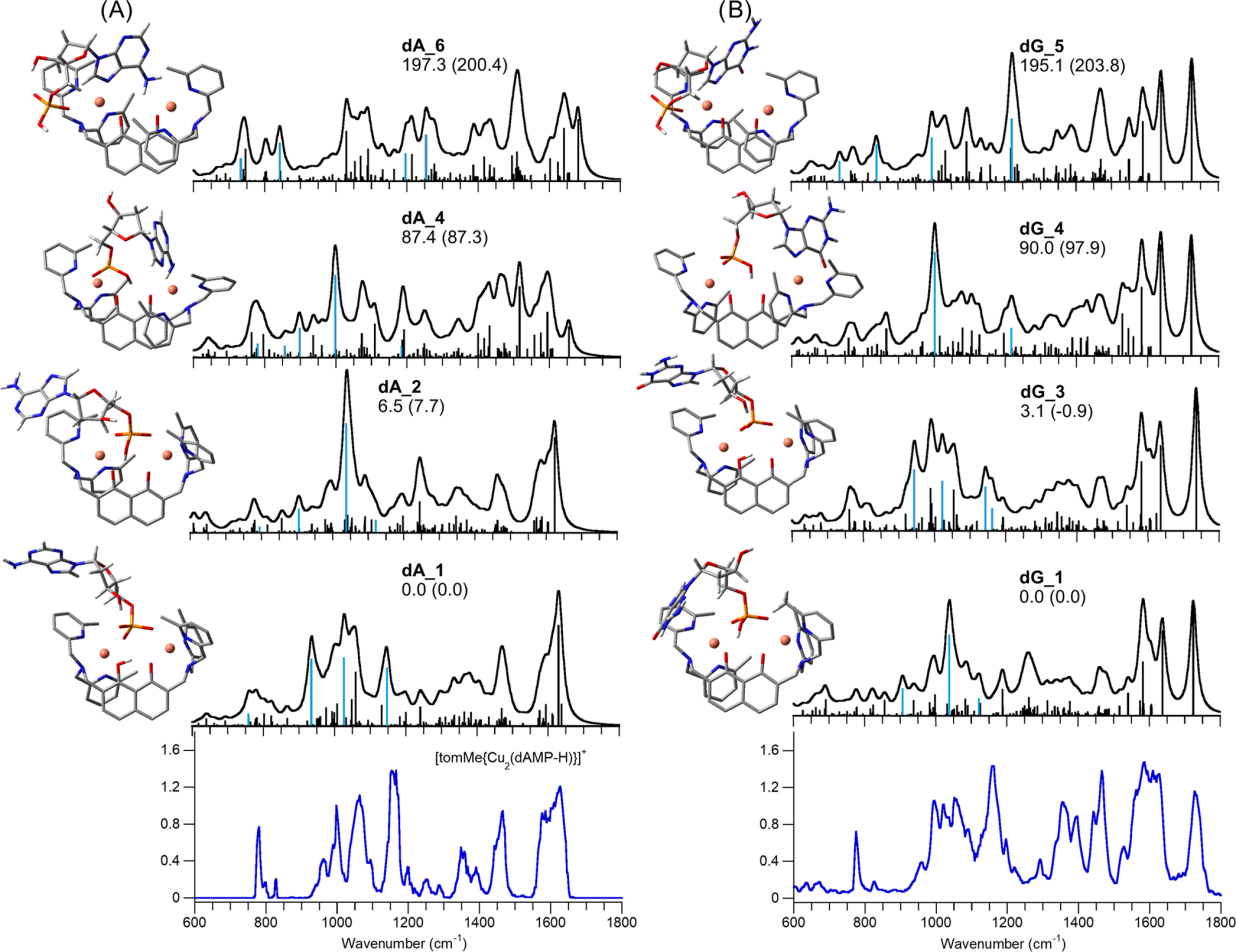
IRMPD spectra of **[1(dAMP-2H)]^+^** (panel (A), bottom) and **[1(dGMP-2H)]^+^** (panel (B), bottom) compared with calculated IR spectra of selected conformers, whose optimized structures are reported on the left of each spectrum. Relative free energies (enthalpies in parentheses) at 298 K are in kJ mol^–1^. Unscaled vibrational modes are highlighted in pale blue.

To confirm the structural features inferred from CID reactivity behavior and gain direct information about the structure of the reactant and product ions, IRMPD spectra were recorded for the sampled ions of interest, namely, **[1(OAc)_2_]^+^**, **[1(HPO_4_)]^+^**, **[1(1,2PO)]^+^**, [**1(1,4PO)]^+^**, **[1(dAMP-2H)]^+^**, and **[1(dGMP-2H)]^+^** at *m*/*z* 881, 859, 951, 979, 1092, and 1108, respectively. These results are illustrated in Figures 3–6, which comprise comparisons of experimental and theoretically predicted IR spectra for selected geometries.

The IRMPD spectrum of **[1(OAc)_2_]^+^** is reported as the blue profile in Figure 3. A few prominent and broad features emerge above 1300 cm^–1^, accompanied by weaker signals in the range below 1200 cm^–1^. A combination of the simulated spectra of the lowest-lying conformers **CuAc2_1**, **CuAc2_2**, and **CuAc2_3** (*G*_rel_^298K^ within 10 kJ mol^–1^, see Table S3 for relative enthalpies and free energies data) can be considered to describe the experimental features. Indeed, the calculated IR spectrum of the highest energy conformer **CuAc2_4** (*G*_rel_ = 36.2 kJ mol^–1^) is in good agreement with the experiment and thus cannot be spectroscopically ruled out from the assayed gas-phase population. However, considering the important energy difference between **CuAc2_4** and conformers **CuAc2_1**–**3**, the following vibrational assignment will be based on the latter ones. In the highest wavenumber range of the experimental spectrum, the broad absorption at 1580 cm^–1^ can be interpreted by a combination of OH bending and CO_2_ asymmetric stretching modes present in all of the conformers in that range. Major bands at 1373 and 1327 cm^–1^ point to the importance of **CuAc2_2** and CuAc2_3, which show strong, calculated absorption bands due to CH_3_ umbrella modes at about 1390 and 1340 cm^–1^. However, the relatively high intensity of the experimental feature at 1447 cm^–1^ confirms the contribution of the lowest-lying isomer **CuAc2_1**, displaying CH_3_ umbrella modes of the acetate ions combined with C=O stretchings in this same spectral range. A detailed description of the vibrational band and mode assignment is reported in Table S4.

All of the lowest-lying conformers **CuAc2_1–3** present small absorptions in the spectral range of interest attributed to different CH bending modes of the pyridine rings (see Table S4), which, summed up, might account for the 1170 cm^–1^ experimental band. As previously described, signal intensities in IRMPD spectra cannot always be directly compared to the calculated vibrational mode activities due to the nonlinearity of the multiple photon dissociation absorption process.^27^

In all calculated structures, each copper ion is coordinated to the nitrogen atoms of the adjacent methylpyridine groups and of the tertiary amine, one of the two oxygen atoms of the dihydroxynaphtalene bridge and both acetate O atoms in a chelate form. Both metals therefore present an octahedral coordination. Among the calculated structures, one can highlight two different families focusing on the position of the bispyridylaminemethyl groups with respect to the dihydroxynaphtalene plane. **CuAc2_1**, **CuAc2_2**, and **CuAc2_3** share the same arrangement with the pyridyl rings A and B on the opposite side of C and D (see Figure 1 for label assignment). This configuration creates a high structural symmetry and reduces the steric tension between the methylpyridine rings. Also, it allows for longer Cu^II^···Cu^II^ distances, i.e., 6.18, 6.12, and 6.07 Å in **CuAc2_1**, **CuAc2_2**, and **CuAc2_3**, respectively, which are values comparable to data stating the metals to be spaced by about 6–7 Å in the crystal structure.^19,20^ On the other hand, in **CuAc2_4**, both pendants are oriented on the same face of dihydroxynaphtalene. Accordingly, the acetate ligands and methylpyridine groups get closer, thus increasing the steric repulsion, which raises the relative free Gibbs energy to 36.2 kJ mol^–1^. It is interesting to note that the distance between copper ions is reduced to 5.92 Å in **CuAc2_4**.

When the two acetate ligands are replaced by a phosphate anion in **[1(HPO_4_)]^+^**, the trend of preferred conformations of the tom^Me^ ligand drastically changes to allow better coordination of phosphate with both copper ions (Figure 4). The lowest-energy structure **PO4_1**, which is ascribed to a [(tom^Me^){Cu_2_(H_2_PO_4_)}]^+^ complex with the extra proton moved on the phosphate ligand, shows a different arrangement of the bispyridylaminemethyl substituents when compared with the lowest-lying diacetate conformer **CuAc2_1**. Indeed, the tom^Me^ conformation of **PO4_1** more closely resembles that of **CuAc2_4** (*G*_rel_^298K^ = 36.2 kJ mol^–1^) with rings A, B and C, D overlooking the same face of the dihydroxynaphtalene group. This configuration allows the copper ions to get closer to each other, as described above, and favors the formation of a bridged-type binding of two oxygen atoms of phosphate with the metal centers. The resulting distance between the copper ions is even shorter, only 5.17 Å, and both metals are penta-coordinate. The phosphate group in **PO4_1** is in the monoanionic form H_2_PO_4_^–^, although one of the protons is oriented toward an oxygen of the deprotonated dihydroxyl moiety forming a strong hydrogen-bond (*r*_POH···O_ = 1.79 Å). Conversely, isomer **PO4_2** shares the same orientation of the rings A, B, C, and D as in **PO4_1** but differs in the charge state of the phosphate, which is doubly deprotonated (HPO_4_^2–^) in the **[1(HPO_4_)]^+^** complex. The proton that is released from the phosphate group is in fact now shared between the two oxygen atoms of the dihydroxynaphtalene unit, allowing the third phosphoryl oxygen to be oriented toward one of the copper ions, leading to a bidentate coordination. **PO4_2** is however higher in Gibbs free energy by 21.2 kJ mol^–1^, suggesting the position of the proton to have an important influence on the energy of the complex. When the HPO_4_^2–^ ligand chelates one copper ion while still binding the other one, the metals get even closer at 4.90 Å. Structure **PO4_3** is based on the Htom^Me^ conformation of **CuAc2_2** and lies 24.9 kJ mol^–1^ above the global minimum. A comparison of the trend in relative free energy for the [Htom^Me^{Cu(OAc)}_2_]^+^ and [(tom^Me^){Cu_2_(HPO_4_)H}]^+^ (where in the latter formula the position of the proton, either on the dihydroxynaphtalene unit or on the phosphate, is left undefined) complexes suggests that (i) when the additional ligand(s) permit the copper ions relatively large spacing, the lowest-lying tom^Me^ conformations present the bispyridylaminemethyl substituents facing opposite directions to reduce steric hindrance (as in **CuAc2_1**, **CuAc2_2**, and **CuAc2_3**); (ii) on the contrary, when a ligand like the phosphate ion restricts the copper ions to get closer to each other, the arrangement of the tom^Me^ ligand is characterized by the molecular pendants oriented on the same dihydroxynaphtalene plane (as in **PO4_1** and **PO4_2**).

The comparison of the IRMPD spectrum with the theoretical IR spectra indicates a major contribution of **PO4_1**, in particular when observing the experimental signals at 1077 and 1050 cm^–1^, which may be assigned to the calculated PO–H bending (not involved in H-bonding) and PO stretching vibrational modes calculated at 1099 and 1051 cm^–1^, respectively. In addition, the envelope of absorptions at 1600 and 1573 cm^–1^ is well simulated by the combination of methylpyridine breathing modes and CC stretching of naphthalene calculated between 1607 and 1573 cm^–1^. Some contribution of **PO4_2** and **PO4_3** can however be envisaged in spite of the higher energy of these conformers viewing the IRMPD band at 1527 cm^–1^, interpreted by the O1H in-plane bending mode calculated at 1523 and 1524 cm^–1^ for **PO4_2** and **PO4_3**, respectively. In addition, the intense experimental band at 1163 cm^–1^ may relate to the calculated features of **PO4_2** and **PO4_3** at 1184 and 1162 cm^–1^, respectively, both assigned to the coupling of a PO stretch and PO–H bending. The intensity of this signal suggests the relative energy of **PO4_2** and **PO4_3** to be somewhat overestimated, probably owing to an inadequate simulation of the position of the added proton by calculations of static structures. It is interesting to note that the use of M06-2X-D3 lowers by more than 10 kJ mol^–1^ both **PO4_2** and **PO4_3** relative free energies, thus producing a better agreement with the spectroscopic evidences. This confirms the necessity of having a proper description of dispersion forces in the calculations of metal complexes interacting with biomolecules. A thorough attribution of experimental bands based on calculations is reported in Table S5, while the whole set of calculated structures and IR spectra is reported in Figure S18.

Figure 5 reports the IRMPD spectra of (A) **[1(1,2PO)]^+^** and (B) **[1(1,4PO)]^+^** compared with the calculated IR spectra of selected conformers. For each complex, three structures (**12PO_1**,**2**,**6** and **14PO_1**,**3**,**7**) were chosen to represent the main binding motifs found for the diphosphonic acids. Figures S19 and S20 show selected optimized structures from two different perspectives for **[1(1,2PO)]^+^** and [**1(1,4PO)**]^+^, respectively. Intriguingly, their relative energy trend is not the same. In panel A, the lowest-lying geometry is ascribed to a [(tom^Me^){Cu_2_(1,2POH)}]^+^ isomer (**12PO_1**), with the tom^Me^ conformation strictly similar to **CuAc2_4** and **PO4_1**. The copper ions are at a distance of 5.16 Å that allows a phosphonic unit to bridge both metals, while the other one can chelate a copper ion, leading to a coordination resembling **PO4_1**. The two binding oxygen atoms of phosphate in **12PO_1** are spaced 5.37 Å apart. Differently, in panel B, the analogous structure [(tom^Me^){Cu_2_(1,4POH)}]^+^ is **14PO_3**, which lies 31.3 kJ mol^–1^ above the global minimum **14PO_1**. The latter geometry, characterized by a Htom^Me^ arrangement very similar to the ones observed in **CuAc2_2** and **PO4_3**, presents the phosphonic groups located at a distance of 7 Å and involved in a chelate interaction, while copper ions are spaced by 5.86 Å. The structure is further stabilized by two intramolecular H-bonds between the phosphonic units (PO–H···OP). The resulting **[1(1,4PO)]^+^** complex (**14PO_1)** is highly symmetrical, with both Cu^II^ ions being six-coordinate and no evident bond length or angle constraints. A different trend holds for panel A, where the lowest-energy **[1(1,2PO)]^+^** structure presenting a proton on the tom^Me^ ligand is **12PO_2**, which lies at 10.3 kJ mol^–1^. This divergence is likely due to the shorter alkyl chain in **1,2PO**, which hinders the formation of the two strong H-bonds observed in **14PO_1** as well as a P=O placement favoring a proper octahedral coordination for one metal ion. Differently, **12PO_6** and **14PO_7** show a single phosphonate unit bound to both copper ions, in an arrangement akin to **PO4_1**, except for the presence of the alkylphosphonic pendant, acting as a peripheral functionality. In agreement with thermodynamic data, the main IRMPD absorptions find predicted counterparts in the bands of the lowest-lying isomers **12PO_1** and **14PO_1** that contribute significantly to the ion population of **[1(1,2PO)]^+^** and **[1(1,4PO)]**^**+**^, respectively. Other identified structures are provided in Figures S21 and S22. Two diagnostic bands in Figure 5 are worth discussing to corroborate the structural assignment. A somewhat intense signal at 1267 cm^–1^ (panel A) is well interpreted by the combination of vibrational modes, including the PO–H bending calculated between 1260 and 1306 cm^–1^ for **12PO_1**, which corresponds to [(tom^Me^){Cu_2_(1,2PO)}H]^+^. Conversely, this mode is barely observed in the experimental profile of panel B, and, consistently, with **14PO_1**, which conforms to a **[1(1,4PO)]^+^** isomer. In addition, the experimental spectrum of the 1,4PO-dicopper complex displays a well-resolved signal at 1523 cm^–1^, which is absent in the spectrum of [(tom^Me^){Cu_2_(1,2PO)}H]^+^. Notably, this band is characteristic of isomers that feature a proton shared by the oxygen atoms of the dihydroxynaphtalene unit, such as **CuAc2_1**-**4**, and **PO4_2**,**3**, and is also present in **14PO_1** at 1521 cm^–1^, corresponding to CO–H bending coupled to CH in-plane bending modes of the naphtalene ring.

The combined experimental and theoretical approach adopted has demonstrated the dicopper complex to favorably interact with two phosphate groups. In particular, the distance of 7 Å between oxygen donors in 1,4-butyldiphosphonic acid ligand enables the copper ions to be spaced by ca. 6 Å. Abiomimetic model complex is thus formed, where the tom^Me^ ligand exposes the methylpyridine groups on opposite faces of the naphthalene plane, thus reducing steric hindrance. This evidence suggests that **[1(OAc)_2_]^+^** might prefer interaction with biological molecules presenting two or more phosphate groups with adequate spacing, as occurring within the nucleic acids, as it was initially intended.^20^

The survey of (model) phosphate-containing ligands has been completed by assaying the dinuclear copper complexes of dAMP and dGMP nucleotides by IRMPD spectroscopy (Figures 6, S23, and S24). The optimized geometries of selected optimized structures of **[1(dAMP-2H)]^+^** and **[1(dGMP-2H)]^+^** are displayed from two different perspectives in Figures S25 and S26, respectively.

The computational analysis performed on the two complexes shows similar geometries that also resemble **[1(HPO_4_)]^+^**, confirming the dicopper complex preference for binding to the phosphate unit. It is possible to identify two isomeric families among the structures with calculated relative free energies lower than 10 kJ mol^–1^. In particular, isomers **dA_2** (*G*_rel_ = 7.7 kJ mol^–1^) and **dG_1** (global minimum) for **[1(dAMP-2H)]^+^** and **[1(dGMP-2H)]^+^**, respectively, show the phosphate group engaged in a bridged interaction with both copper ions, while the remaining POH is involved in a H-bond interaction with the O atoms of the deprotonated dihydroxynaphtalene. Conformers **dA_3** and **dG_2** (*G*_rel_ = 8.3 and 2.3 kJ mol^–1^, respectively) pertain to the same family, both differing only for the nucleoside orientation, which allows the sugar hydroxyl group of both ions to interact with the POH oxygen atom, hence resembling the most stable conformers already described for bare deprotonated nucleotides.^68^ Indeed, the use of the functional M06-2X-D3 highlights the stabilization induced by the π–π interaction between the methylpyridine rings of tom^Me^ and the nucleobases, characteristic of **dA_2** and **dG_1**, as compared to the H-bond interaction between deoxyribose and the phosphate oxygen present in **dA_3** and **dG_2**. Differently, using both B3LYP and B3LYP-D3, **dA_3** and **dG_2** geometries are lower in free energies than **dA_2** and **dG_1**, respectively (Table S3). Structures **dA_1** and **dG_3** (*G*_rel_ = 0.0 and 3.1 kJ mol^–1^, respectively) show a comparable binding motif as **PO4_2**, with a chelate coordination on one copper and a single contact on the second, while the remaining proton resides on the dihydroxynaphtalene unit (Htom^Me^). Interestingly, these isomers get progressively lower in relative free energies moving from B3LYP to B3LYP-D3 and finally M06-2X-D3 (Table S3). Structures endowed with either (i) coordination at the copper ions by both the phosphate and N7 site of the nucleobase, i.e., **dA_4** and **dG_4** or (ii) binding at either metal ion by the nucleobase unit without any contact involving the phosphate group, i.e., **dA_6** and **dG_5**, are much less favorable (*G*_rel_ > 85 kJ mol^–1^) and may thus be energetically ruled out. A comprehensive presentation of other conceivable higher energy structures is provided in Figures S21 and S22. Noteworthily, all of the lowest-lying isomers, **dA_1–3** and **dG_1–3**, display the phosphate moiety interacting with both copper ions, in agreement with tandem mass spectrometry evidence and previous reports.^19,20,23,24^ Although the bond strength of copper ions to phosphates is weaker than a metal–nucleobase interaction,^69^ the rational design of the present dicopper complex enables a selective molecular recognition for the phosphate moiety, offering an alternative binding mode with respect to cisplatin-based drugs.

The IRMPD spectra of **[1(dAMP-2H)]^+^** and **[1(dGMP-2H)]^+^** show many similarities, particularly below 1500 cm^–1^, where intense bands at 1467, 1357, 1157, 1065, and 995 cm^–1^ are shared. In the higher wavenumber range, both experimental spectra present a broad absorption between 1587 and 1623 cm^–1^, but **[1(dGMP-2H)]^+^** is also characterized by two bands at 1730 and 1530 cm^–1^, likely due to the different nucleobase. An admixture of the lowest-energy **dA_1–3** and **dG_1–3** isomers can be invoked to account for the experimental spectra of the dicopper complexes with dAMP and dGMP, respectively. One vibrational mode of particular interest to elucidate the binding motif of the nucleotides is the PO_2_ asymmetric stretching calculated at ca. 1045 cm^–1^ for both **dA_2,3** and **dG_1**,**2**, in agreement with the IR activity of both experimental spectra in that range. The high intensity of the band at 1157 cm^–1^ in both IRMPD spectra suggests a significant contribution to the assayed ion populations due to isomers **dA_1** and **dG_3**, the only ones presenting a P=O stretching mode involved in H-bonding with the dihydroxynaphtalene group, predicted at 1147 and 1143 cm^–1^, respectively. While **dA_2,3** and **dG_1**,**2** correspond to a [(tom^Me^){Cu_2_(dA(G)MP-H)}]^+^ adduct, isomers **dA_1** and **dG_3**, are ascribed to a [**1(dA(G)MP-2H)**]^+^ complex with the proton residing on the dihydroxynaphtalene ligand.

In the higher energy range, characteristic bands of **[1(dGMP-2H)]^+^** are in agreement with the C=O stretching and the ring breathing modes of guanine nucleobase calculated at 1734 and 1544 cm^–1^ for **dG_1**. The NH_2_ scissoring mode is instead calculated for both **dA_1** and **dG_1** at ca. 1630 cm^–1^ in agreement with the strong absorption observed at 1623 cm^–1^. This band shows pronounced broadening toward the red, well simulated by a combination of purine CH bending modes that are accompanied by the NH bending of guanine at 1583 cm^–1^ in the **dG_1-4** isomers. To summarize, both thermodynamic and spectroscopic data point to the predominant presence of isomers in which the phosphate group interacts with the copper ions of the [tom^Me^Cu_2_]^2+^ complex. Indeed, although a cooperative interaction of both the phosphate and the nucleobase with the copper ions cannot be completely excluded through the participation of **dA_4** and **dG_4** isomers, the preference for the complex to engage in mere phosphate–dinuclear copper coordination, by sampling **dA_1–3**, and **dG_1–3**, is unambiguous.

## Conclusions

The cytotoxic activity toward human cancer cells of a novel family of dinuclear Cu^II^ and Ni^II^ complexes, developed to bind selectively to two neighboring phosphate esters of DNA backbone, was established before by in vitro cytotoxicity assays backed by spectroscopic, biochemical, and single-molecule experiments. However, regarding the dinuclear Cu^II^ complexes, an unambiguous identification of their binding motifs with nucleic acids was not yet obtained in the condensed phase.

In the present contribution, a detailed description of the binding motifs of the dinuclear Cu^II^ complex obtained by ligand exchange of [(HtomMe){Cu(OAc)}_2_]^+^ with representative phosphate-containing ligands, L, ranging from inorganic phosphate up to mononucleotides, i.e., dAMP and dGMP, has been gathered, supporting the potential of this promising complex endowed with anticancer activity to target DNA via a new binding mode with respect to cisplatin. **[1(L-2H)]^+^** adducts formed in solution were successfully delivered to the gas phase by ESI through the substitution of two coordinated acetate ligands. Unambigous evidence of direct Cu-phosphate contacts has been obtained by a combination of tandem mass spectrometry, IRMPD spectroscopy in the fingerprint region, and quantum chemical calculations. The dissociation channels observed by CID and IRMPD assay display the direct loss of the coordinated ligand only for acetate and phosphate, whereas the diphosphonate and nucleotide adducts undergo only a prominent fragmentation of the tom^Me^ skeleton, in agreement with a strong interaction of the phosphate functionality to both copper ions.

The molecular structure of **[1(HPO_4_)]^+^** is represented by a mixture of the two lowest-energy isomers, where inorganic phosphate bridges the two Cu^II^ ions, and the position of the added proton is either on the dihydroxynaphtalene unit or on a phosphate oxygen. A similar coordination is assigned to the nucleotide complexes **[1(dAMP-2H)]^+^** and **[1(dGMP-2H)]^+^**. Both species participate in the sampled ion population as two isomeric forms which share the same phosphate/Cu_2_^II^ coordination but again differ in the position of a proton, which is either located on the phosphate ligand or on tom^Me^ phenol functionality. Accordingly, both spectroscopy and calculations assess that competitive binding of the dinuclear Cu^II^ complex to the nucleobase is inhibited. In **[1(1,2PO)]^+^** and **[1(1,4PO)]^+^**, where bridged diphosphonates act as a good mimic of the DNA backbone, spectral evidence reveals the sole presence of the lowest-lying isomers, in which both phosphonic groups are interacting with the copper ions. In particular, in **[1(1,2PO)]^+^**, a phosphate bridges the two copper ions, while the other chelates one Cu^II^ ion; in **[1(1,4PO)]^+^**, both phosphate groups form a bidentate interaction with each of the copper ions, assessing the possibility of the complex to bind proximal phosphate groups in the DNA backbone, as originally intended.

Overall, although some solution-phase structural features may be preserved downstream of the ESI process, as suggested, for example, by the survival of metal-ligands noncovalent complexes, the removal of the solvent may enhance the role of electrostatic intramolecular interactions. Thus, considerations based only on thermodynamics may not be suitable, since higher energy species formed in solution, where they are instead favored by solvation, can be kinetically trapped in the gas phase and survive the electrospray ionization, thus contributing to the sampled population together with low-lying gas-phase isomers. The influence of solvent molecules on either zwitterionic or nonzwitterionic structures of (metalated) amino acids has been largely investigated by theory and experiment, and IRMPD spectroscopy has provided a direct and reliable tool to determine the number of water molecules required to stabilize the zwitterion form.^70−72^

The results obtained here may therefore be regarded as circumstantial evidence that **[1(L-2H)_2_]^+^** is coordinated to the phosphate groups in the bare dicopper–ligand complexes.

## Supporting Information Available

Retarding potential analyses (Figure S1); ESI FT-ICR mass spectra (Figures S2, S6, S8, and S12); CID mass spectra (Figures S3 and S7); breakdown curves (Figures S4, S9, and S15); photofragmentation mass spectrum (Figure S5); putative dissociation pattern (Figures S10, S11, S16, and S17); ESI-mass spectra (Figures S13 and S14); IRMPD spectra compared with calculated IR spectra (Figures S18–S26); accurate and exact mass values (Table S1); phenomenological dissociation threshold energies (Table S2); calculated thermodynamic values (Table S3); and experimental and computed IR bands (Tables S4 and S5) (PDF)
