# Supplementary material for: Binding Modes of a Cytotoxic Dinuclear Copper(II) Complex with Phosphate Ligands Probed by Vibrational Photodissociation Ion Spectroscopy
Source: Inorg Chem. 2023 Jan 19;62(4):1341–53. doi: 10.1021/acs.inorgchem.2c02091 (PMC9890465; doi:10.1021/acs.inorgchem.2c02091)
Supplement: Supplementary file 1 — ic2c02091_si_001.pdf [file ic2c02091_si_001.pdf]

Supporting Information

Binding modes of a cytotoxic dinuclear copper(II) complex with phosphate ligands probed by vibrational photodissociation ion spectroscopy

Marco Giampà,[1] Davide Corinti,[2,*] Alessandro Maccelli,[2] Simonetta Fornarini,[2] Giel Berden,[3] Jos Oomens,[3] Sabrina Schwarzbich,[4] Thorsten Glaser,[4] Maria Elisa Crestoni[2, *]

[1]Department of Clinical and Molecular Medicine, Norwegian University of Science and Technology, Olav Kyrres Gate 9, 7030 Trondheim, Norway

[2]Dipartimento di Chimica e Tecnologie del Farmaco, Università di Roma "La Sapienza", I-00185 Roma, Italy

[3]Radboud University, Institute for Molecules and Materials, FELIX Laboratory, Toernooiveld 7, 6525ED Nijmegen, the Netherlands

[4]Lehrstuhl für Anorganische Chemie I, Fakultät für Chemie, Universität Bielefeld, D-33615 Bielefeld, Germany

Figure S1. Retarding potential analyses for [**1(OAc)$_2$**]$^+$ (m/z 881, Panel A), [**1(HPO$_4$**)]$^+$ (m/z 859, Panel B), [**1(1,2PO**)]$^+$ (m/z 951, Panel C) and [**1(1,4PO**)]$^+$ (m/z 979, Panel D).

Figure S2. ESI FT-ICR mass spectrum of [**1(OAc)$_2$**]$^+$. The insert shows the comparison between the experimental (red profile) and the theoretical (black profile) isotopic distribution.

Figure S3. CID mass spectrum of [**1(OAc)$_2$**]$^+$ (m/z 881) recorded at Elab = 20 eV (panel A); fragmentation pathways for dissociation of ions at m/z 881 and putative structure of fragment ions (panel B).

Figure S4. Relative abundances of mass selected **[1(OAc)$_2$]$^+$** ions and product ions as a function of collision energy (center of mass) during CID assay, and extrapolation of the fragmentation onset to determine the phenomenological threshold energy (TE) value.

Figure S5. Photofragmentation mass spectrum of **[1(AcO)$_2$]$^+$** at m/z 881 recorded after irradiation at 1600 cm^{-1}.

Figure S6. ESI(+) FT-ICR mass spectrum of [**1(HPO$_4$**)]$^+$ observed at m/z 859.15156.

Figure S7. CID mass spectrum of [**1(HPO$_4$**)]$^+$ (m/z 859) recorded at Elab = 40 eV (panel A); fragmentation pattern for m/z 859 and putative structure of fragment ions (panel B).

Figure S8. ESI(+) FT-ICR mass spectrum of **[1(1,2PO)]$^+$** (*m/z* 951) and **[1(1,4PO)]$^+$** (*m/z* 979) in panel A and B, respectively.

Figure S9. Relative abundances of: A) mass selected [**1(1,2PO**)]$^+$ ions (blue profile, m/z 951) and product ions (red profile, m/z 724); B) [**1(1,4PO**)]$^+$ (blue profile, m/z 979) and product ions (red profile, m/z 752), as a function of collision energy (center of mass) during CID assay.

Figure S10. CID mass spectrum of **[1(1,2PO)]$^+$** (m/z 951) recorded at Elab = 50 eV (panel A); dissociation pattern of **[1(1,2PO)]$^+$** with putative structures of fragment ions is reported in panel B.

.

Figure S11. CID mass spectrum of **[1(1,4PO)]**$^+$ (m/z 979) recorded at Elab = 30 eV (panel A); dissociation pattern of **[1(1,4PO)]**$^+$ with putative structures of fragment ions is reported in panel B.

Figure S12. ESI(+) FT-ICR mass spectrum of [**1(dAMP-2H**)]$^+$ (*m/z* 1092) and [**1(dGMP-2H**)]$^+$ (*m/z* 1108) in panel A and B, respectively. The inserts show the comparison between the experimental (red profile) and the theoretical (black profile) isotopic distribution.

Figure S13. ESI(+) mass spectrum of [**1(dAMP-2H**)]$^+$ (m/z 1092-1096) and [**1(dGMP-2H**)]$^+$ (m/z 1108-1112).

Figure S14. CID mass spectra of A) [**1(dAMP-2H**)]$^+$ (m/z 1092) recorded at Elab = 0.40 a.u. and B) [**1(dGMP-2H**)]$^+$ (m/z 1108) recorded at Elab = 10 a.u.

Figure S15. Relative abundances of mass selected A) **[1(dAMP-2H)]**$^+$ and B) **[1(dGMP-2H)]**$^+$ ions and product ions as a function of collision energy (center of mass) during CID assay, and extrapolation of the fragmentation onset to determine the phenomenological threshold energy (TE) value.

Figure S16. Putative dissociation pattern of [**1(dAMP-2H]**$^+$ (m/z 1092).

Figure S17. Putative dissociation pattern of [**1(dGMP-2H**)]$^+$ (m/z 1108).

Figure S18. IRMPD spectrum of **[1(HPO4)]**$^+$ (bottom panel) compared with calculated IR spectra of conformers and isomers, whose optimized structures are reported on the left. Relative free energies (enthalpies) at 298 K in kJ mol^{-1}.

Figure S19. Optimized structures of **12PO_1**, **12PO_2** and **12PO_6** each from two different perspectives.

Figure S20. Optimized structures of **14PO_1**, **14PO_3** and **14PO_7** each from two different perspectives.

Figure S21. IRMPD spectrum of **[1(1,2PO)]**$^+$ (bottom panel) compared with calculated IR spectra of conformers and isomers, whose optimized structures are reported on the left. Relative free energies (enthalpies) at 298 K in kJ mol^{-1}.

Figure S22. IRMPD spectrum of **[1(1,4PO)]**$^+$ (bottom panel) compared with calculated IR spectra of conformers and isomers, whose optimized structures are reported on the left. Relative free energies (enthalpies) at 298 K in kJ mol^{-1}.

Figure S23. IRMPD spectrum of **[1(dAMP-2H)]**$^+$ (bottom panel) compared with calculated IR spectra of conformers and isomers, whose optimized structures are reported on the left. Relative free energies (enthalpies) at 298 K in kJ mol^{-1}.

Figure S24. IRMPD spectrum of **[1(dGMP-2H)]**$^+$ (bottom panel) compared with calculated IR spectra of conformers and isomers, whose optimized structures are reported on the left. Relative free energies (enthalpies) at 298 K in kJ mol^{-1}.

Figure S25. Optimized structures of **dA_1**, **dA_2**, **dA_4** and **dA_6** each from two different perspectives.

Figure S26. Optimized structures of **dG_1**, **dG_2**, **dG_4** and **dG_5** each from two different perspectives.

Table S1. Accurate (m_{acc}) and exact mass (m_{ex}) values of the sampled species detected by high-resolution ESI(+) FT-ICR MS experiments from the reaction of **[1(OAc)$_2$]$^+$** with phosphate ligands.

Table S2. Phenomenological threshold energies for the dissociation channels -L and -$C_{14}H_{17}N_3$ of [1(OAc)$_2$]$^+$, [1(HPO$_4$)]$^+$, [1(1,2PO)]$^+$, [1(1,4PO)]$^+$, [1(dAMP-2H)]$^+$, and [1(dGMP-2H)]$^+$ ions.

Table S3. Thermodynamic data for the optimized structures of **[1(OAc)$_2$]$^+$**, **[1(HPO$_4$)]$^+$**, **[1(1,2PO)]$^+$**, **[1(1,4PO)]$^+$**, **[1(dAMP-2H)]$^+$** and **[1(dGMP-2H)]$^+$**.

Table S4. IRMPD resonances and theoretical IR frequencies (cm^{-1}), infrared absorption intensities (km mol^{-1}) in parenthesis, of **CuAc2_1**, **CuAc2_2**, **CuAc2_3**, **CuAc2_4** and vibrational normal modes obtained from DFT calculations at the B3LYP level.

Table S5. IRMPD resonances and theoretical IR frequencies (cm^{-1}), infrared absorption intensities (km mol^{-1}) in parenthesis, of **PO4_1**, **PO4_2** and vibrational normal modes obtained from DFT calculations at the B3LYP level.

Retarding potential analysis

Retarding potential analyses have been conducted by means of a hybrid triple-quadrupole linear ion-trap (2000 Q TRAP, Applied Biosystem) mass spectrometer with a $Q1q2Q_{LIT}$ configuration. The center-of-mass frame has been calculated according to Equation S1

$$E^{CM} = (CE + EP - 0N)\frac{m}{(m + M)} \quad [S1]$$

where CE corresponds to the collision energy applied between Q1 and q2 quadrupoles; EP represents the entrance potential; 0N is the nominal zero collision energy; while m and M are the masses of the collision gas (N_2) and of the mass-selected ion, respectively.

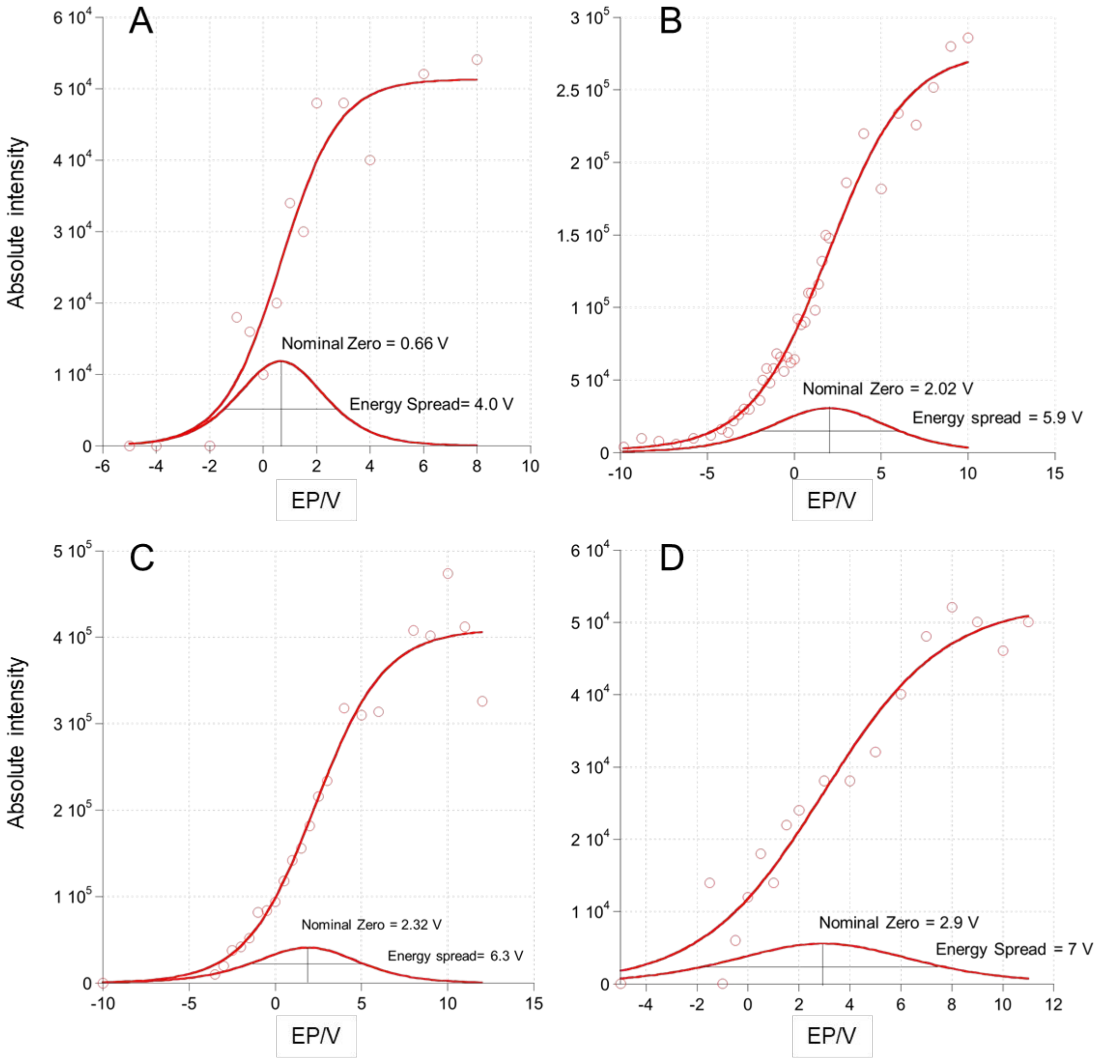

Figure S1. Retarding potential analyses of **[1(OAc)$_2$]**$^+$ (m/z 881, Panel A), **[1(HPO$_4$)]**$^+$ (m/z 859, Panel B), **[1(1,2PO)]**$^+$ (m/z 951, Panel C) and **[1(1,4PO)]**$^+$ (m/z 979, Panel D). The energy resolutions of the kinetic energy are 4.0 ± 0.1 V, 5.9 ± 0.1 V, 6.3 ± 0.1 V and 7.0 ± 0.1 V in the laboratory frame (full width at half maximum, determined from three independent measurements), for m/z 881, 859, 951 and 979, respectively. In the same manner, nominal zero values are obtained at 0.66 V for **[1(OAc)$_2$]**$^+$, at 2.02 V for **[1(HPO$_4$)]**$^+$, at 2.32 V for **[1(1,2PO)]**$^+$ and at 2.9 V for **[1(1,4PO)]**$^+$ ions.

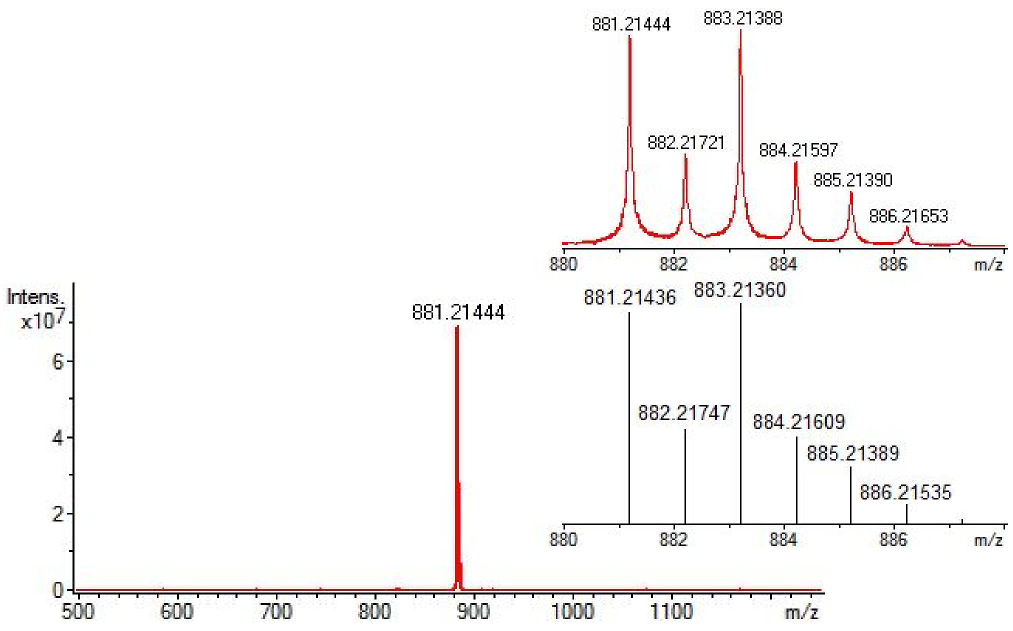

Figure S2. ESI FT-ICR mass spectrum of **[1(OAc)$_2$]$^+$**. The insert shows the comparison between the experimental (red profile) and the theoretical (black profile) isotopic distribution.

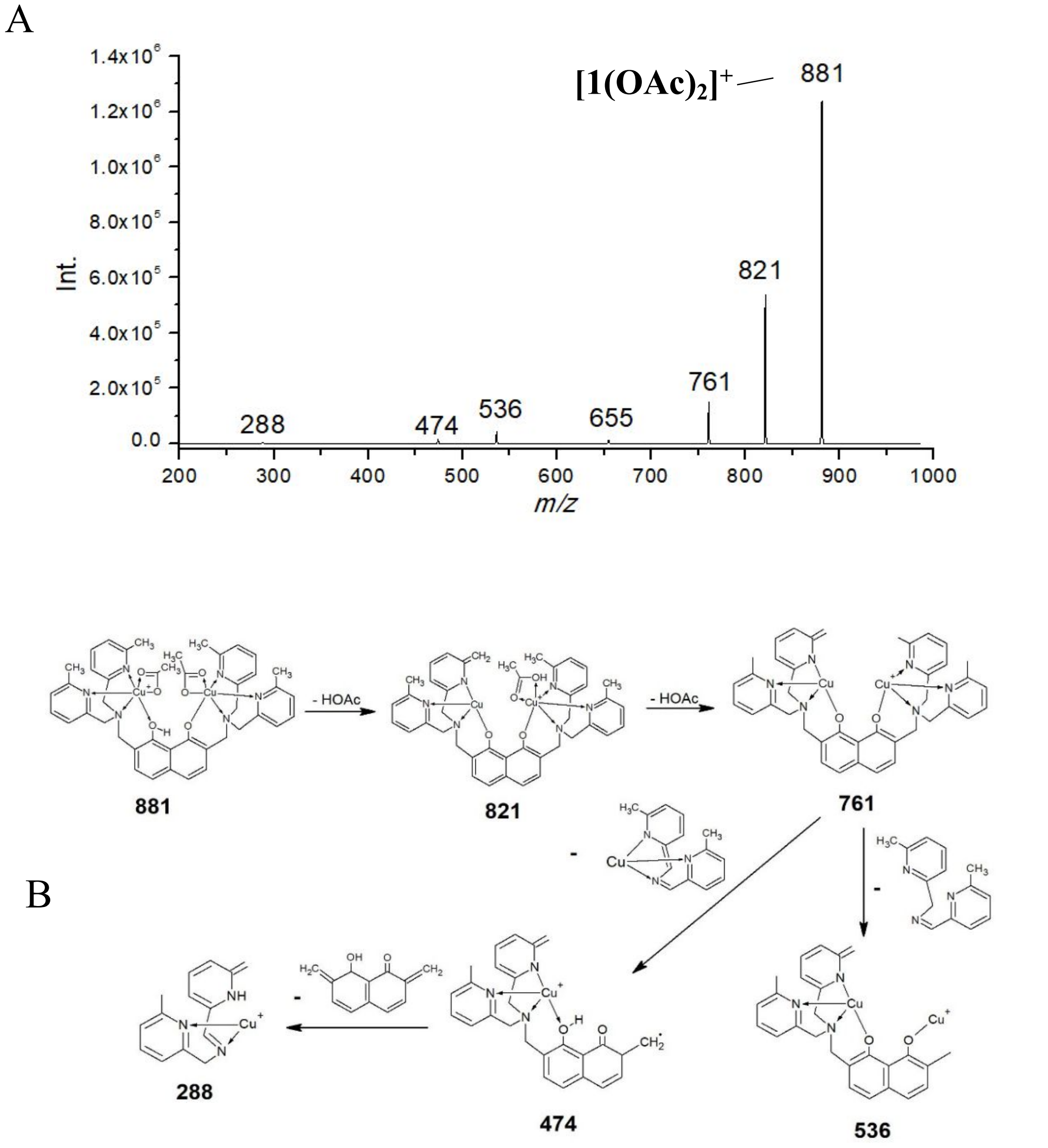

Figure S3. CID mass spectrum of **$[1(OAc)_2]^+$** (m/z 881) recorded at Elab = 20 eV (panel A); fragmentation pattern of ions at m/z 881 and putative structure of fragment ions (panel B).

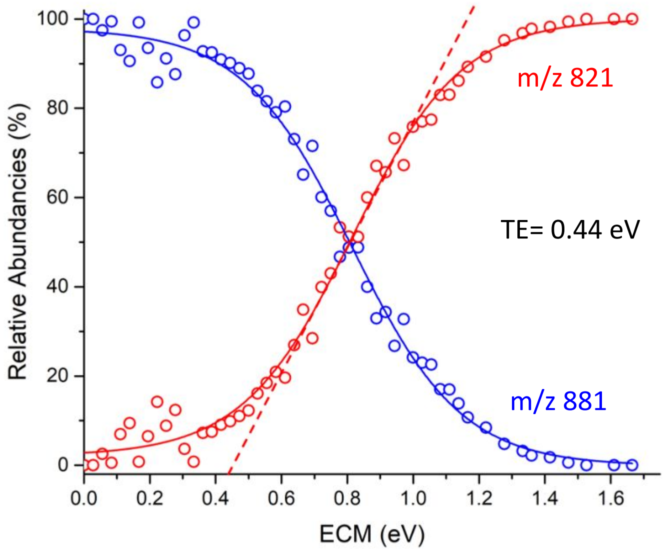

Figure S4. Relative abundances of mass selected **[1(OAc)$_2$]$^+$** ions (blue profile, *m/z* 881) and product ion (red profile, m/z 821) as a function of collision energy (center of mass) during CID assay and extrapolation of the fragmentation onset to determine the phenomenological threshold energy (TE) value.

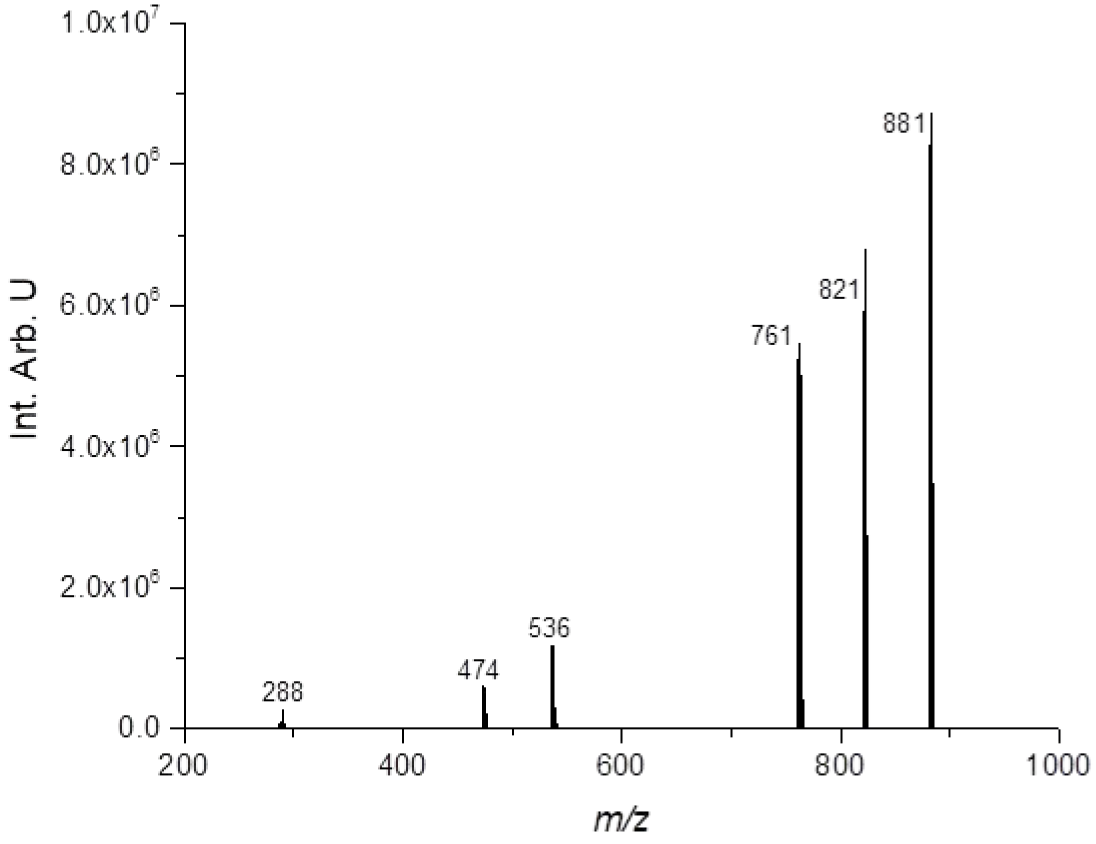

Figure S5. Photofragmentation mass spectrum of **[1(AcO)$_2$]$^+$** at m/z 881 recorded after irradiation at 1600 cm^{-1}.

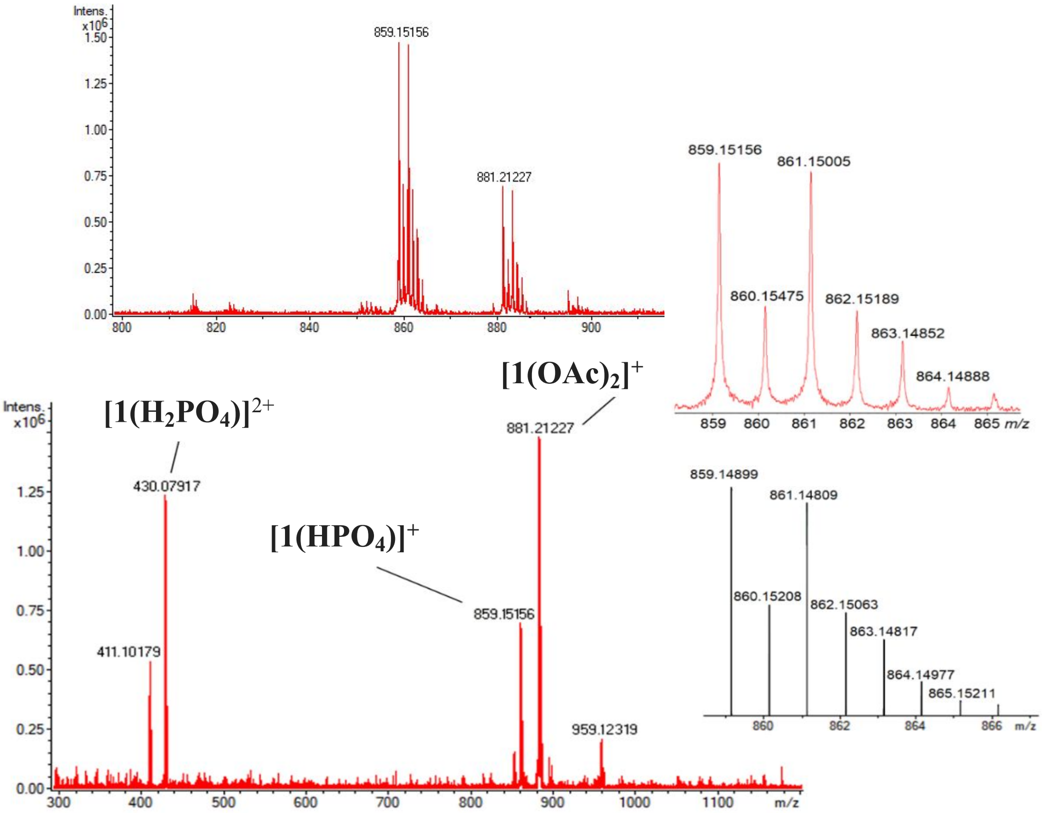

Figure S6. ESI(+) FT-ICR mass spectrum of [**1(HPO$_4$)**]$^+$ observed at m/z 859.15156. The insert shows the comparison between the experimental (red profile) and theoretical (black profile) isotopic distribution.

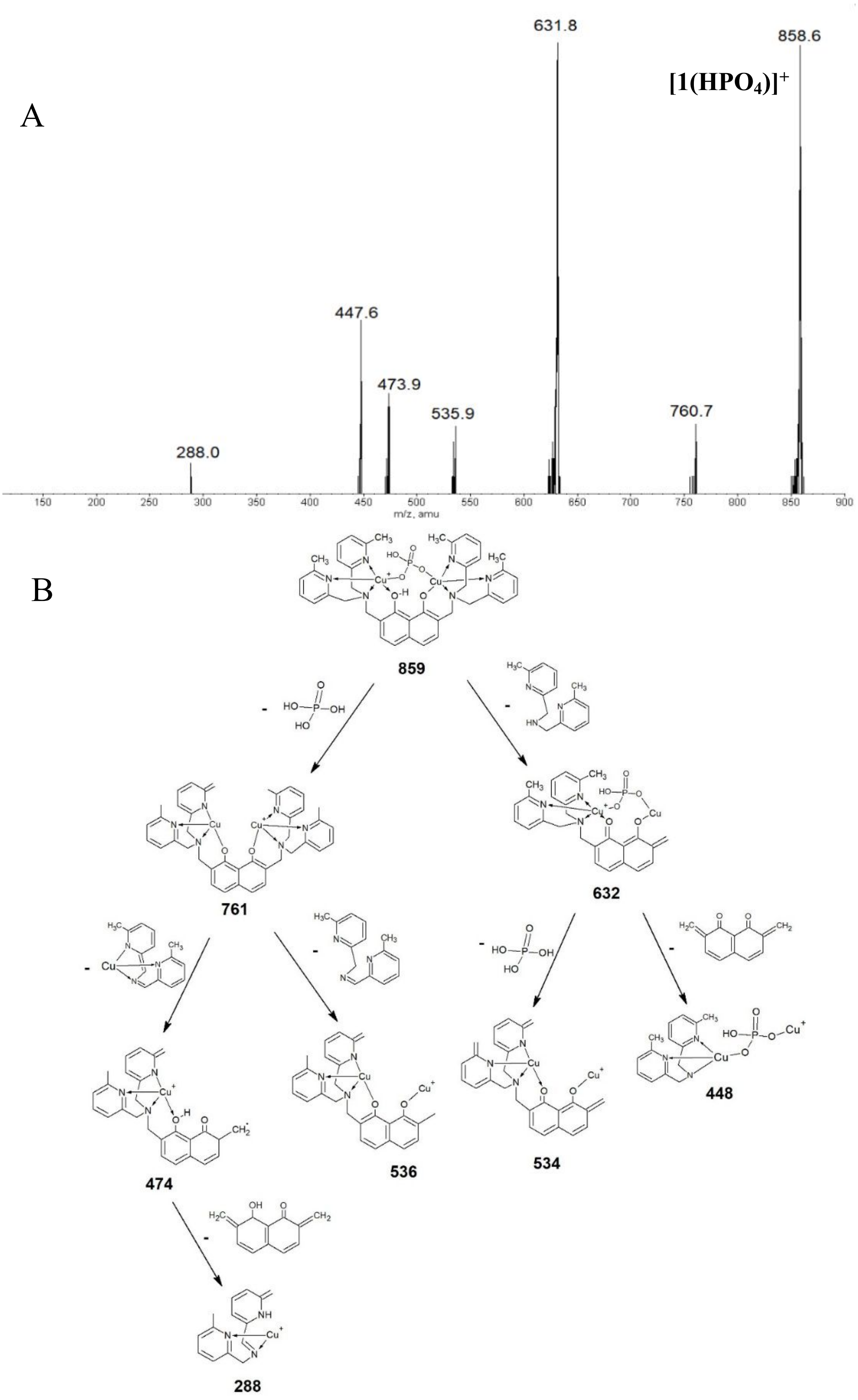

Figure S7. CID mass spectrum of [**1(HPO$_4$)**]$^+$ (m/z 859) recorded at Elab = 40 eV (panel A); fragmentation pattern for m/z 859 and putative structure of fragment ions (panel B).

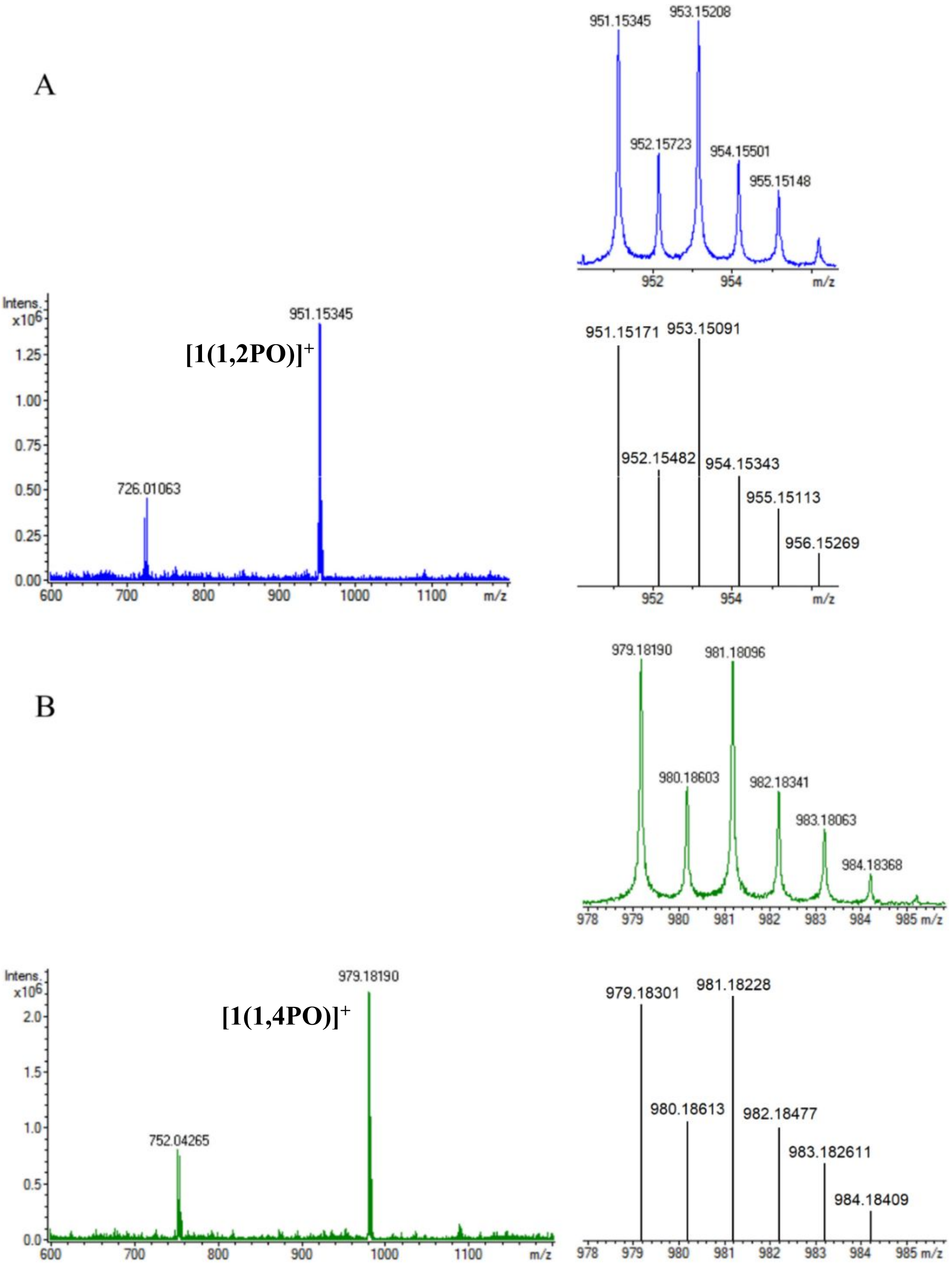

Figure S8. ESI(+) FT-ICR mass spectrum of **[1(1,2PO)]**$^+$ (*m/z* 951) and **[1(1,4PO)]**$^+$ (*m/z* 979) in panel A and B, respectively. The inserts show the comparison between the experimental (blue/green profile) and the theoretical (black profile) isotopic distribution.

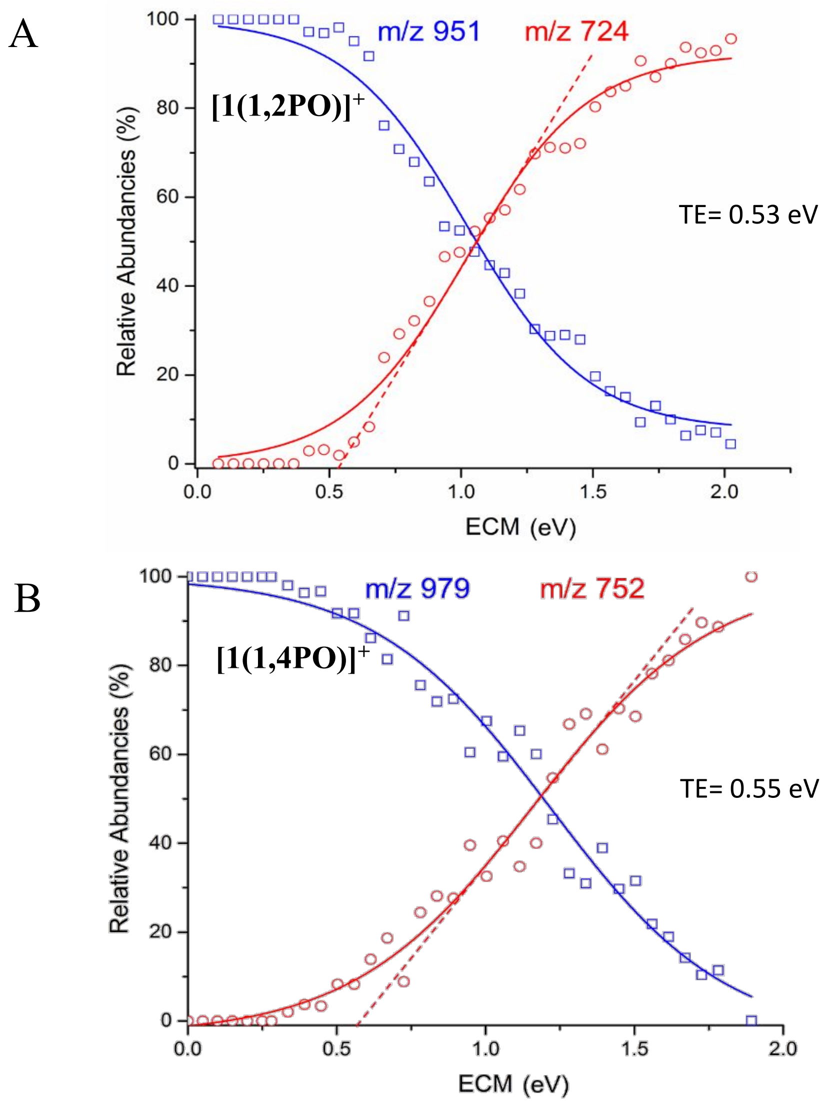

Figure S9. Relative abundances of: A) mass selected [**1(1,2PO)**]$^+$ ions (blue profile, *m/z* 951) and product ions (red profile, m/z 724); B) [**1(1,4PO)**]$^+$ (blue profile, *m/z* 979) and product ions (red profile, m/z 752), as a function of collision energy (center of mass) during CID assay. The extrapolation of the fragmentation onset allows to determine the threshold energy (TE) value (see Table S2).

A

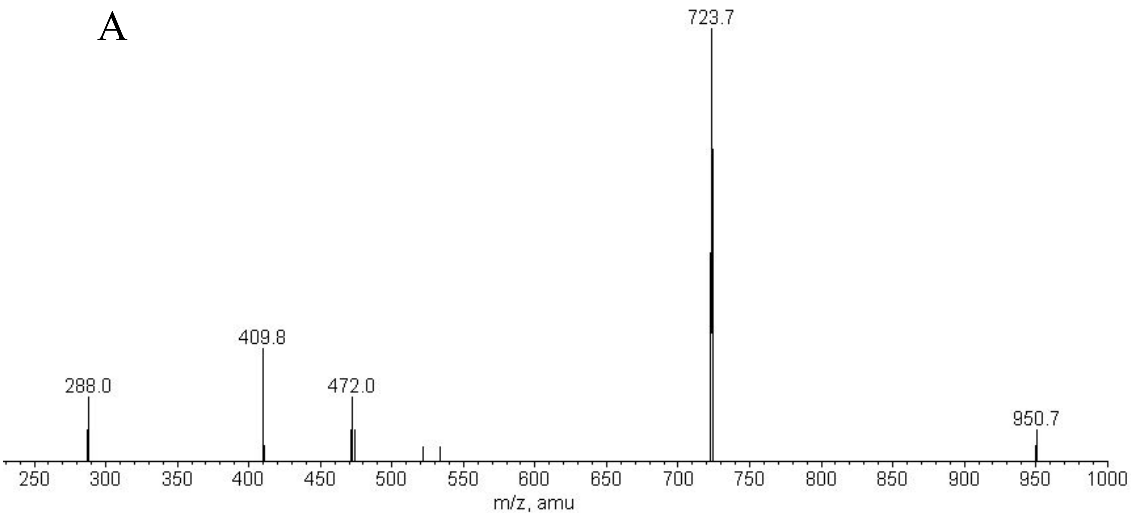

B

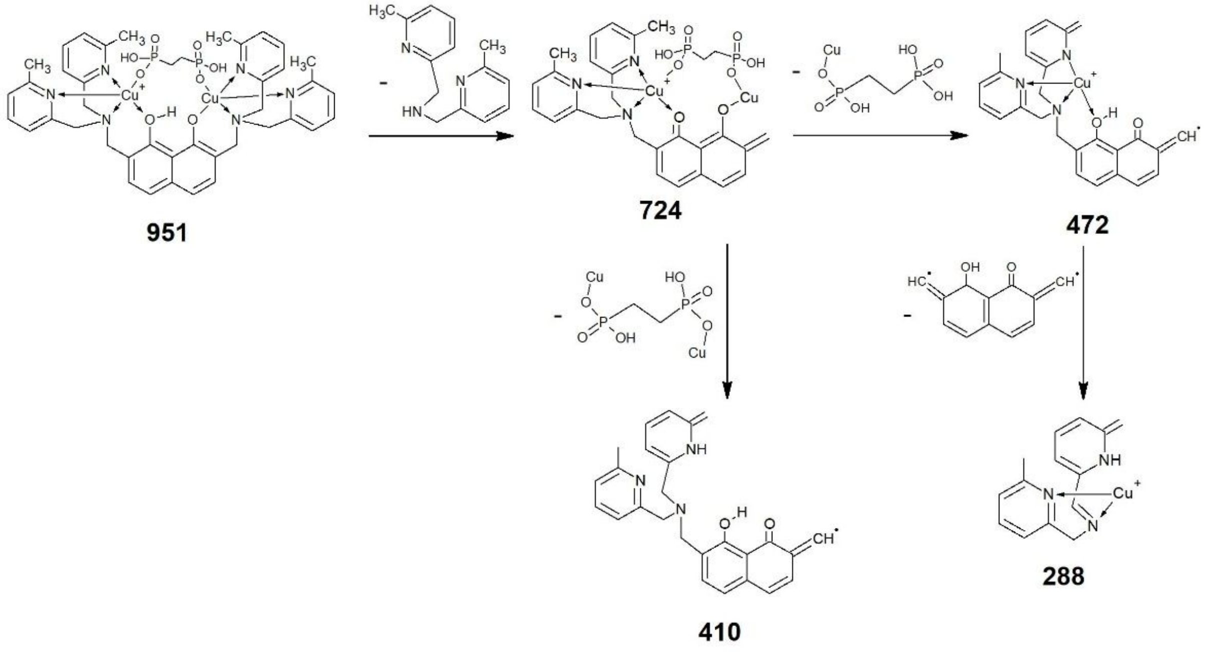

Figure S10. CID mass spectrum of **[1(1,2PO)]$^+$** (m/z 951) recorded at Elab = 50 eV (panel A); dissociation pattern of **[1(1,2PO)]$^+$** with putative structures of fragment ions is reported in panel B.

A

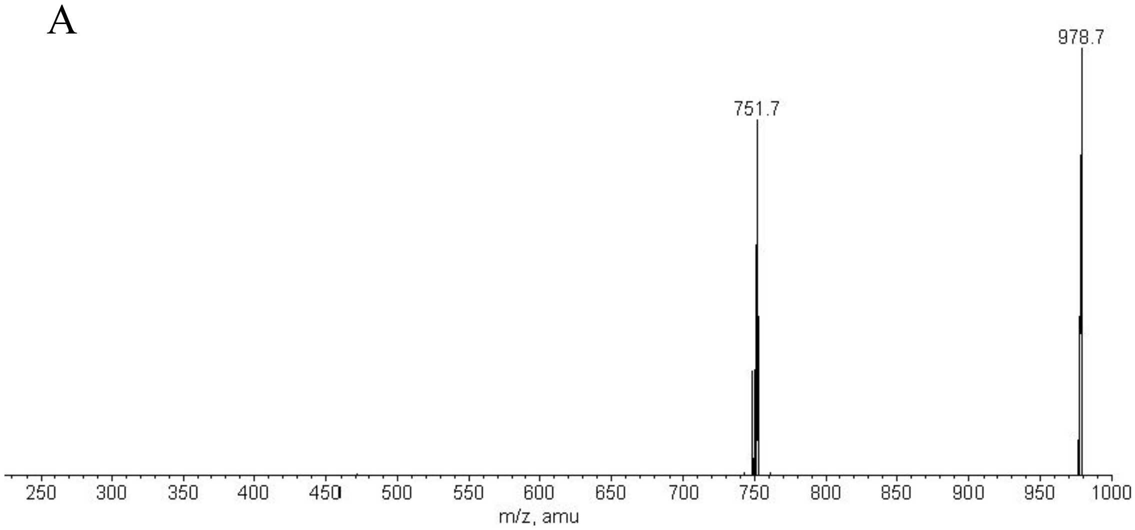

B

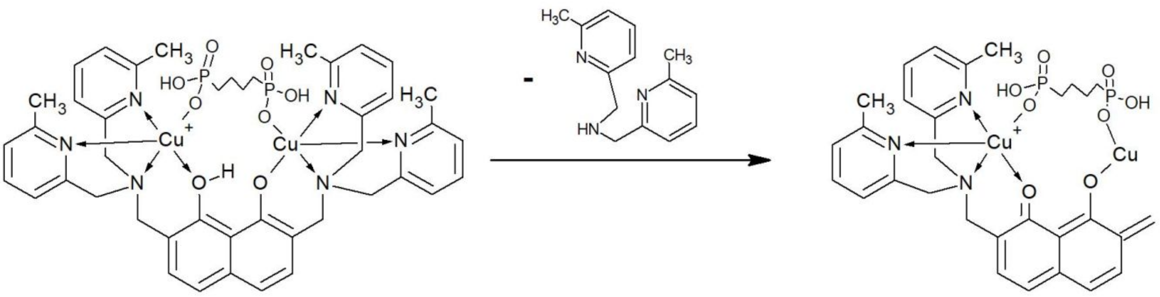

979 **752**

Figure S11. CID mass spectrum of **[1(1,4PO)]**$^+$ (m/z 979) recorded at Elab = 30 eV (panel A); dissociation pattern of **[1(1,4PO)]**$^+$ with putative structures of fragment ions is reported in panel B.

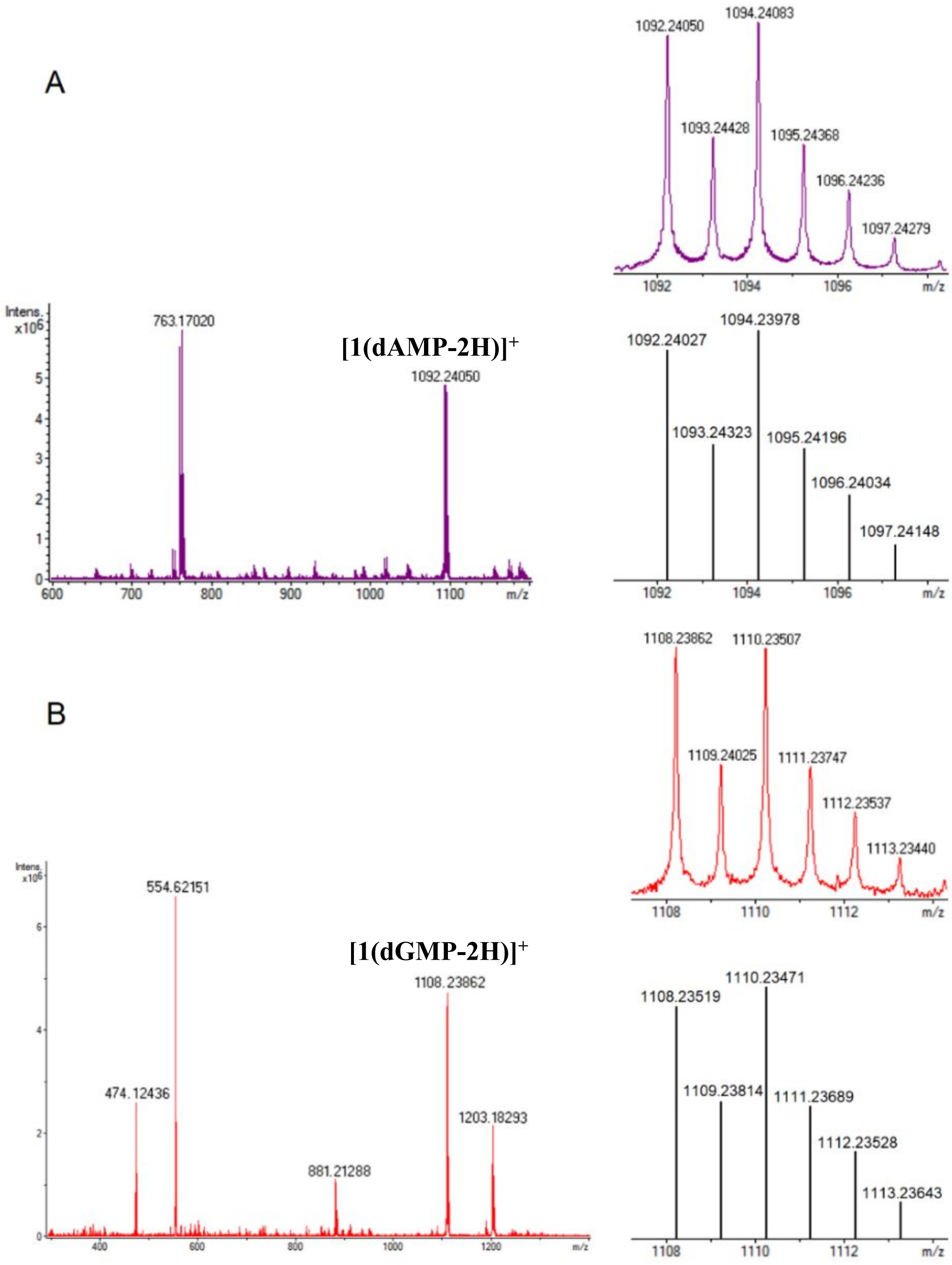

Figure S12. ESI(+) FT-ICR mass spectrum of [**1(dAMP-2H)**]$^{+}$ (*m/z* 1092) and [**1(dGMP-2H)**]$^{+}$ (*m/z* 1108) in panel A and B, respectively. The inserts show the comparison between the experimental (violet or red profile) and the theoretical (black profile) isotopic distribution.

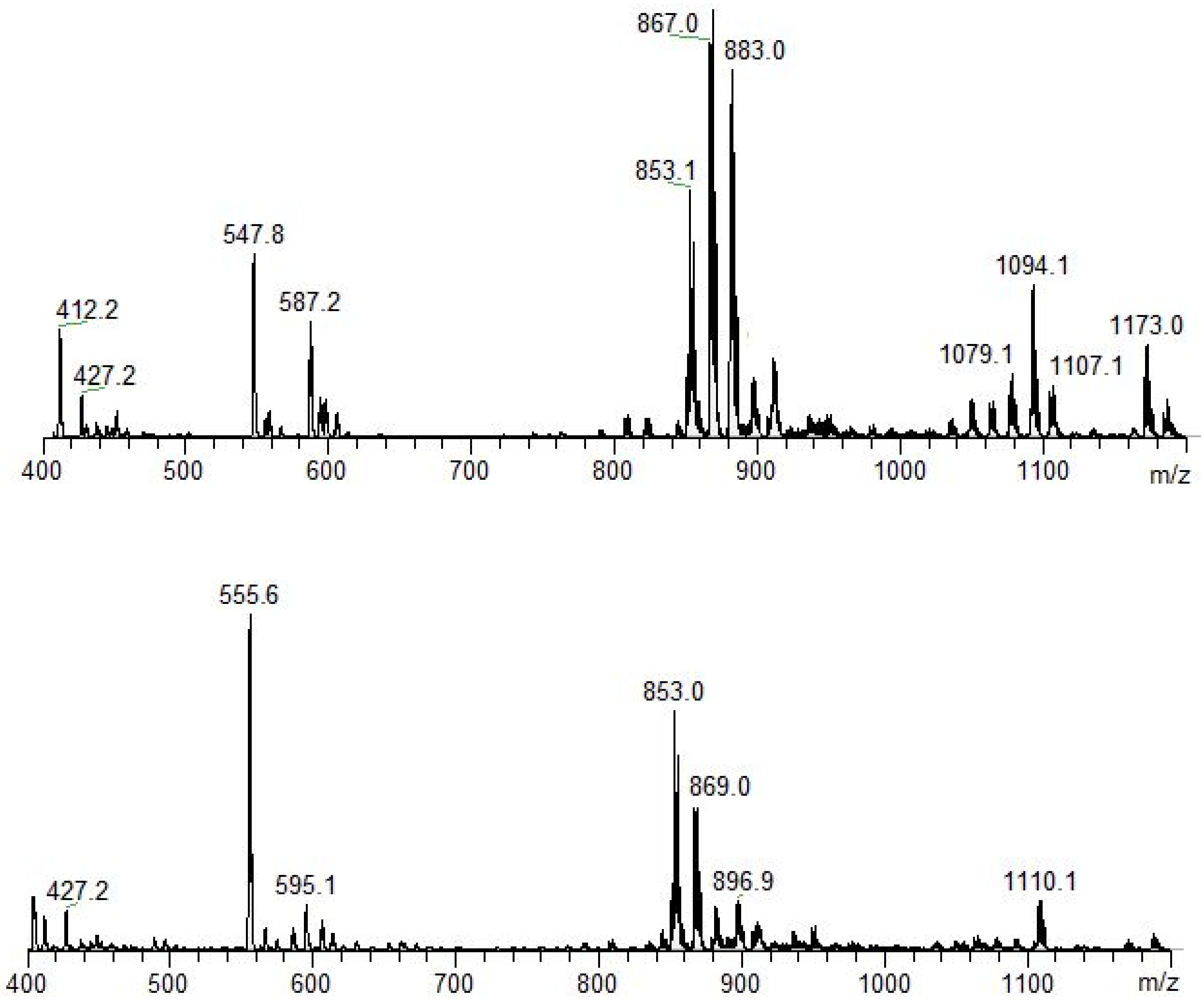

Figure S13. ESI(+) mass spectrum of [**1(dAMP-2H)**]$^+$ (m/z 1092-1096) and [**1(dGMP-2H)**]$^+$ (m/z 1108-1112).

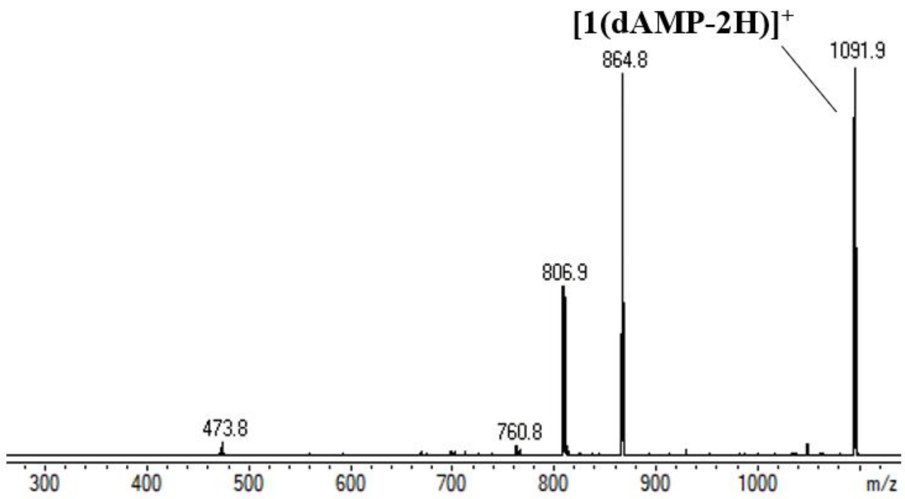

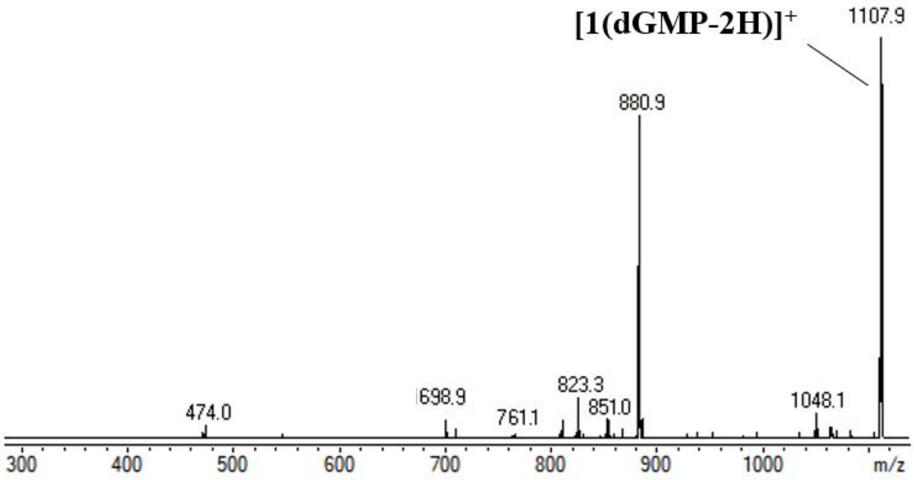

Figure S14. CID mass spectra of A) [**1(dAMP-2H**)]$^+$ (m/z 1092) recorded at Elab = 0.40 a.u. and B) [**1(dGMP-2H**)]$^+$ (m/z 1108) recorded at Elab = 10 a.u.

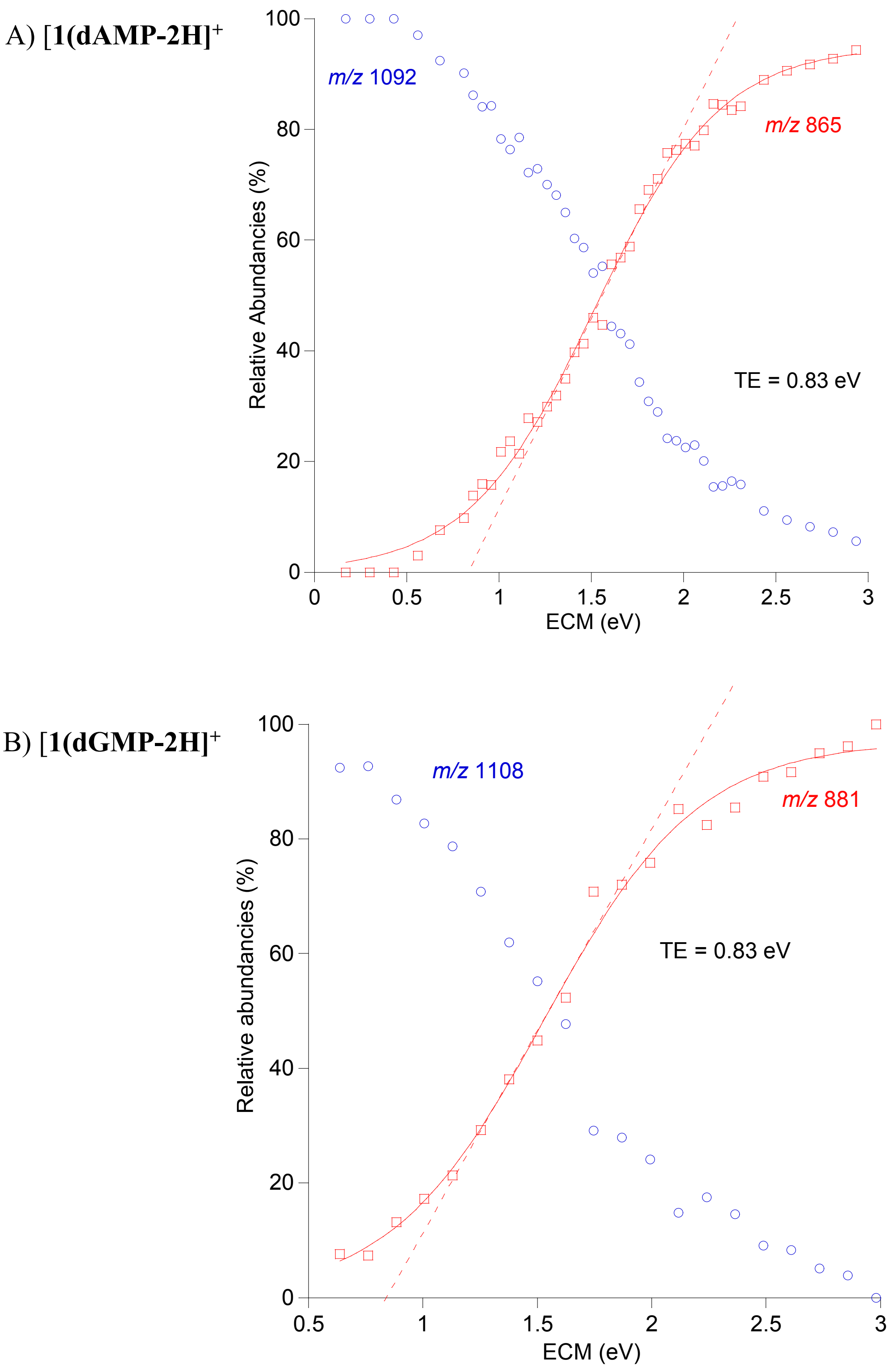

Figure S15. Relative abundances of mass selected A) **[1(dAMP-2H)]**$^+$ and B) **[1(dGMP-2H)]**$^+$ ions and product ions as a function of collision energy (center of mass) during CID assay, and extrapolation of the fragmentation onset to determine the phenomenological threshold energy (TE) value.

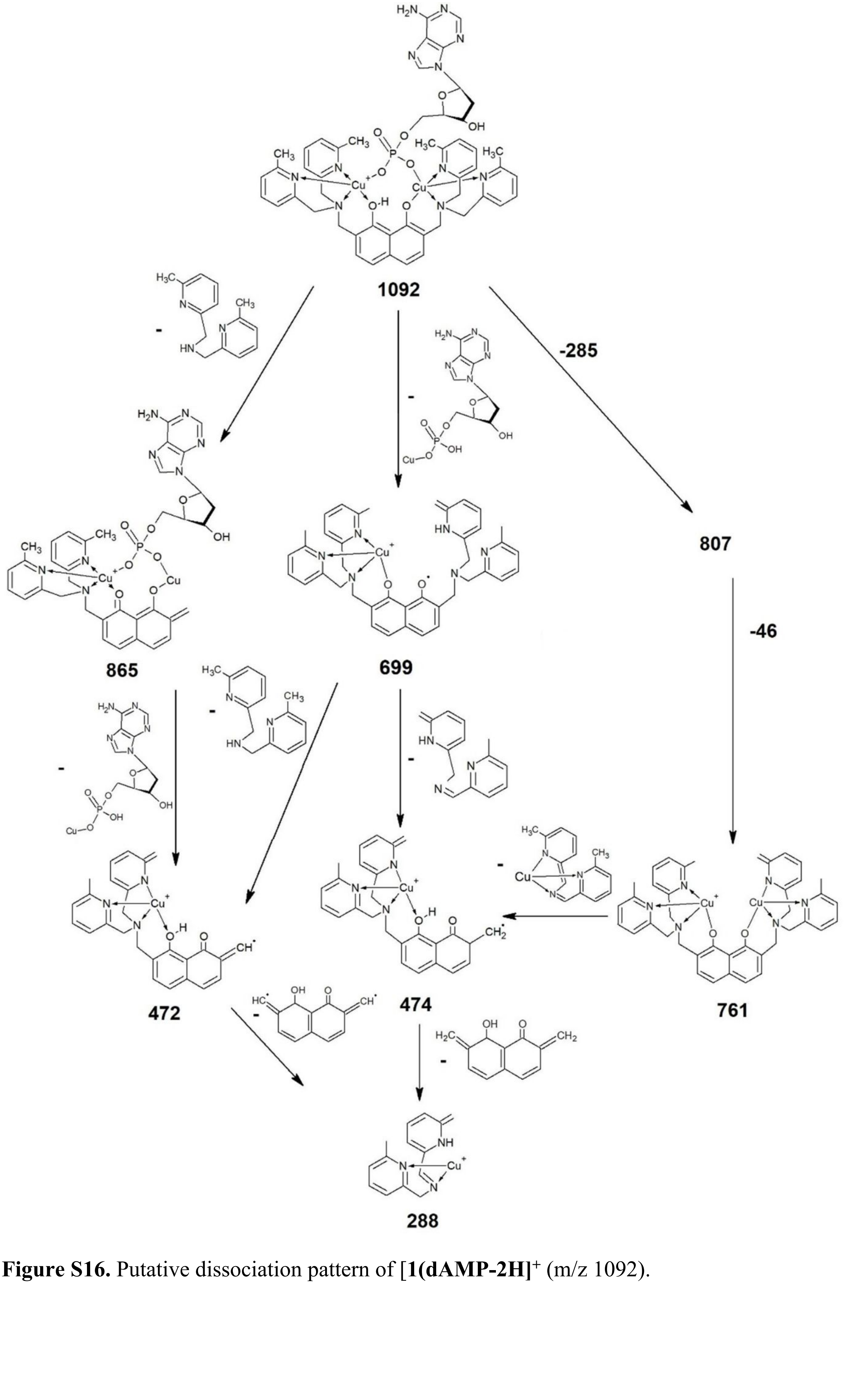

Figure S16. Putative dissociation pattern of [**1(dAMP-2H]**$^+$ (m/z 1092).

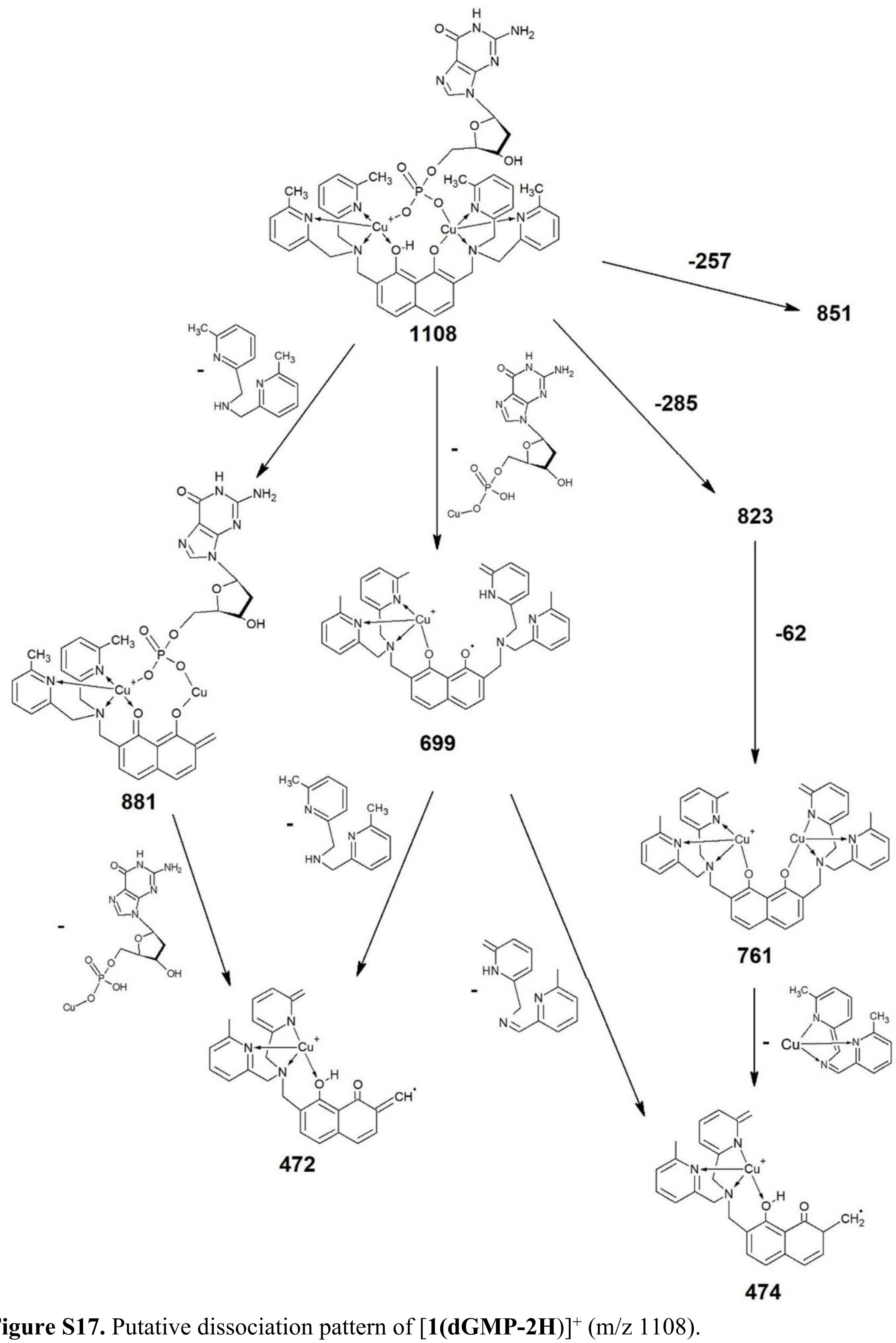

Figure S17. Putative dissociation pattern of [**1(dGMP-2H**)]$^+$ (m/z 1108).

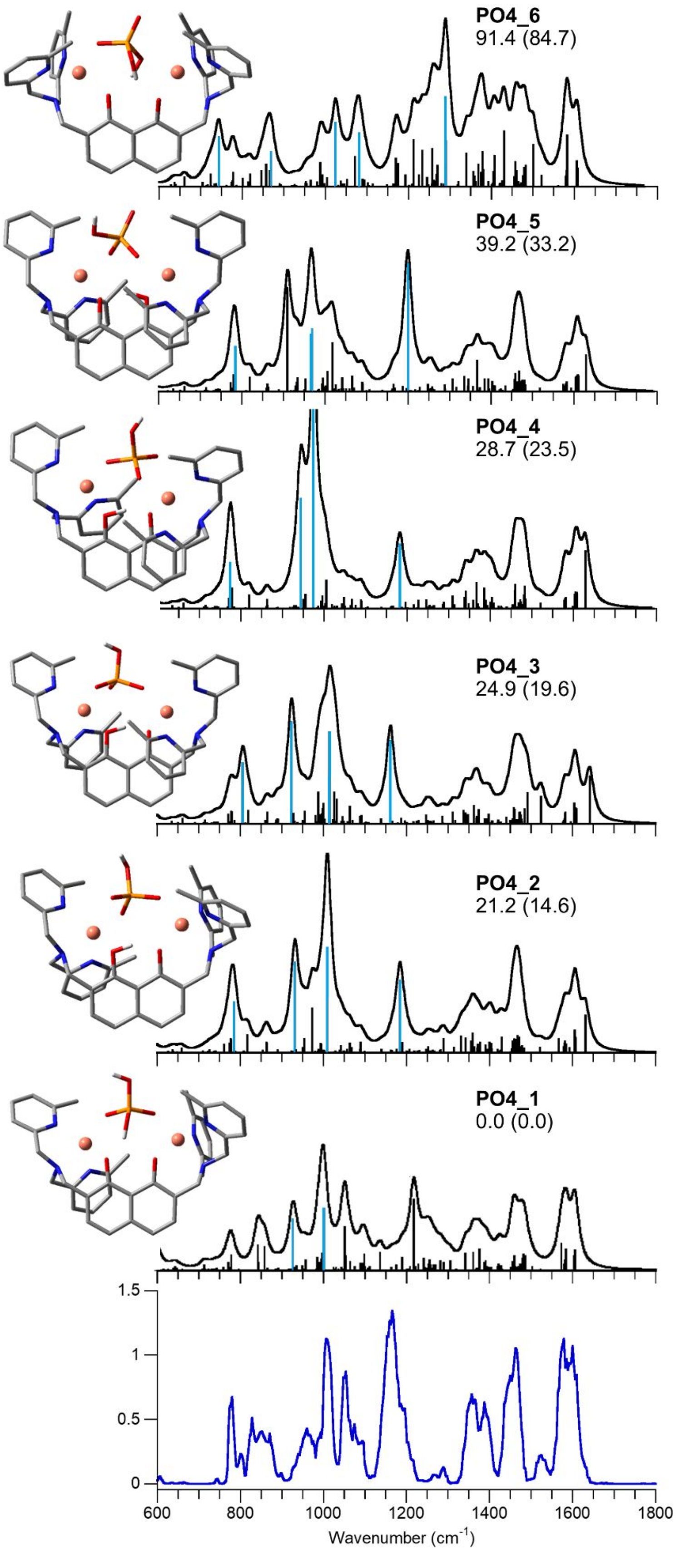

Figure S18. IRMPD spectrum of **[1(HPO4)]**$^+$ (bottom panel) compared with calculated IR spectra of conformers and isomers, whose optimized structures are reported on the left. Relative free energies (enthalpies) at 298 K in kJ mol^{-1}. Unscaled vibrational modes are highlighted in pale blue.

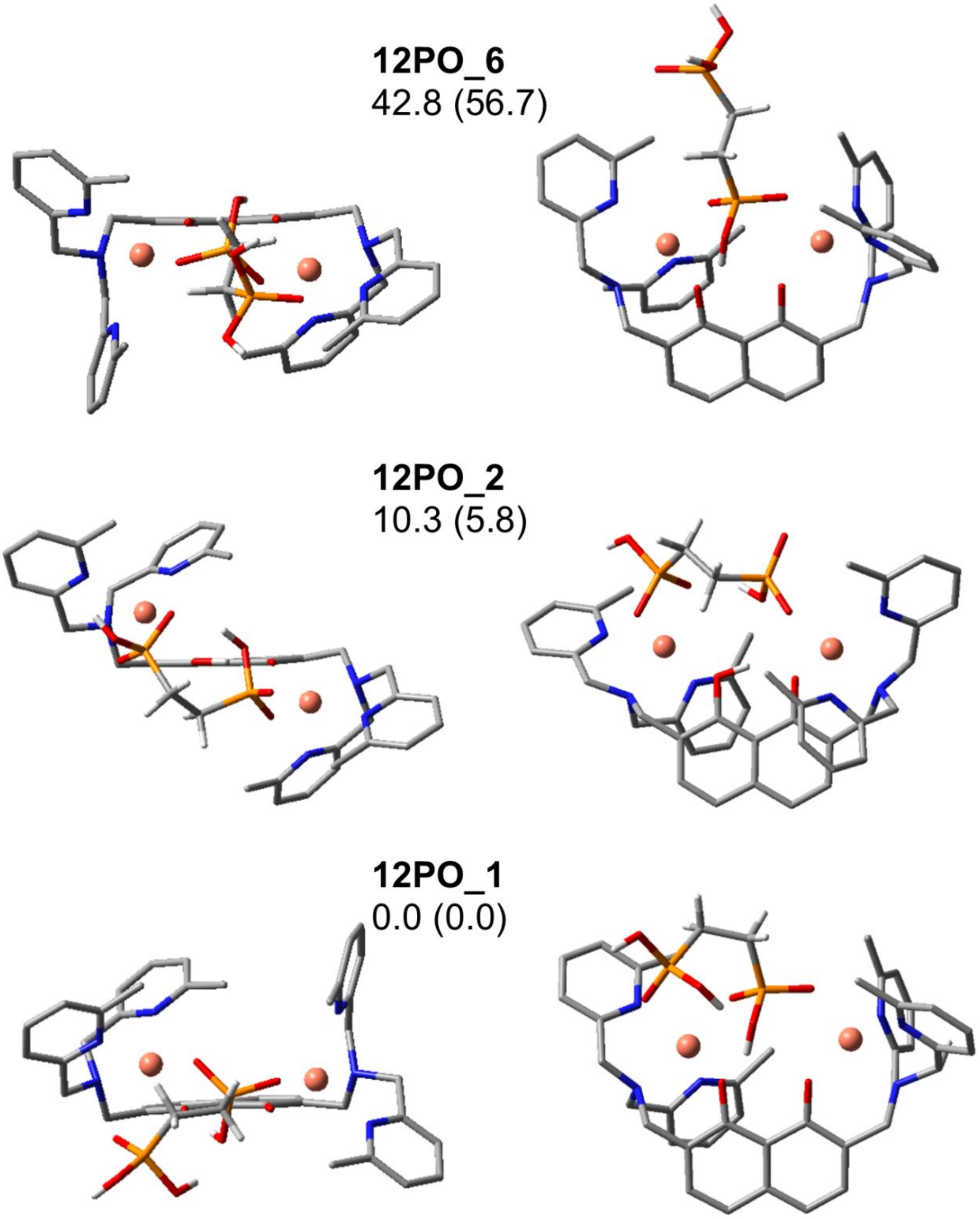

Figure S19. Optimized structures of **12PO_1**, **12PO_2** and **12PO_6** each from two different perspectives.

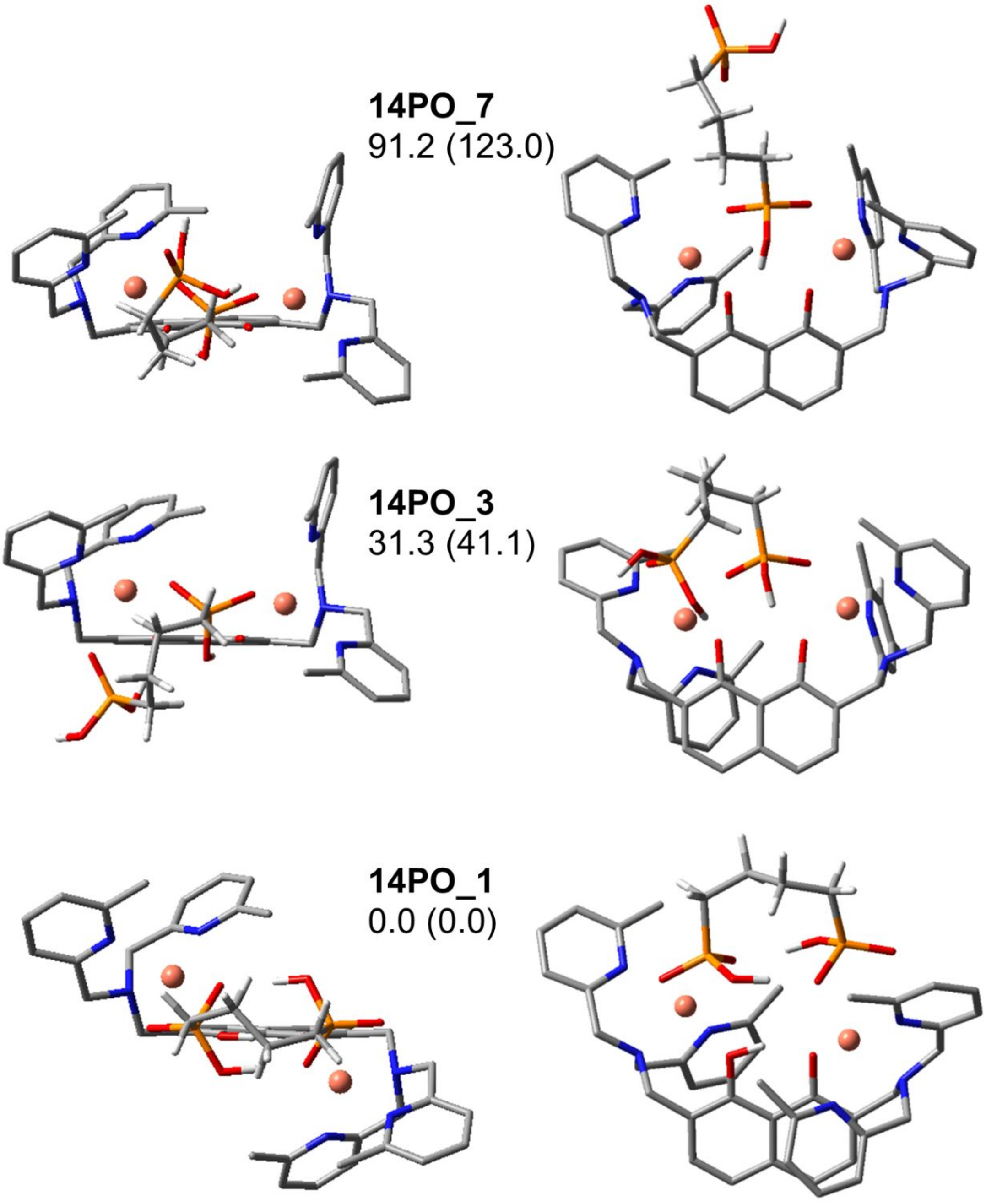

Figure S20. Optimized structures of **14PO_1**, **14PO_3** and **14PO_7** each from two different perspectives.

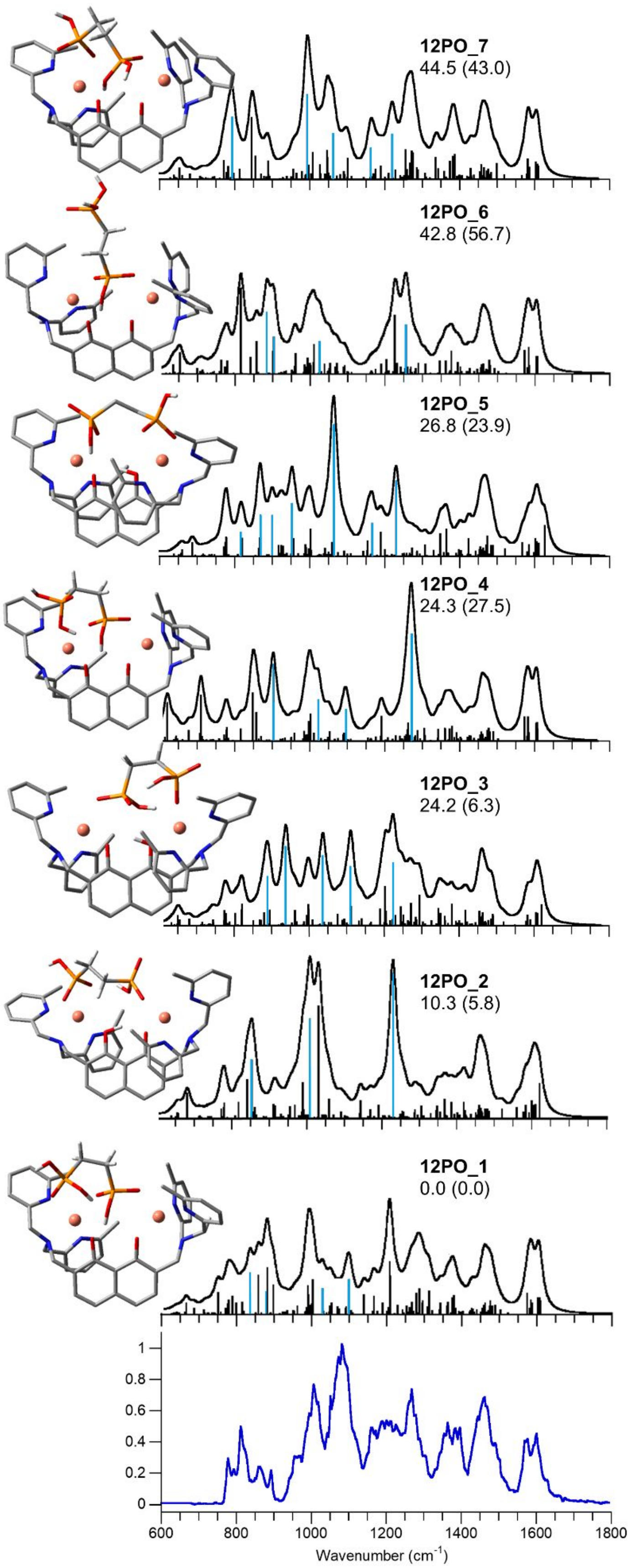

Figure S21. IRMPD spectrum of **[1(1,2PO)]**$^+$ (bottom panel) compared with calculated IR spectra of conformers and isomers, whose optimized structures are reported on the left. Relative free energies (enthalpies) at 298 K in kJ mol^{-1}. Unscaled vibrational modes are highlighted in pale blue.

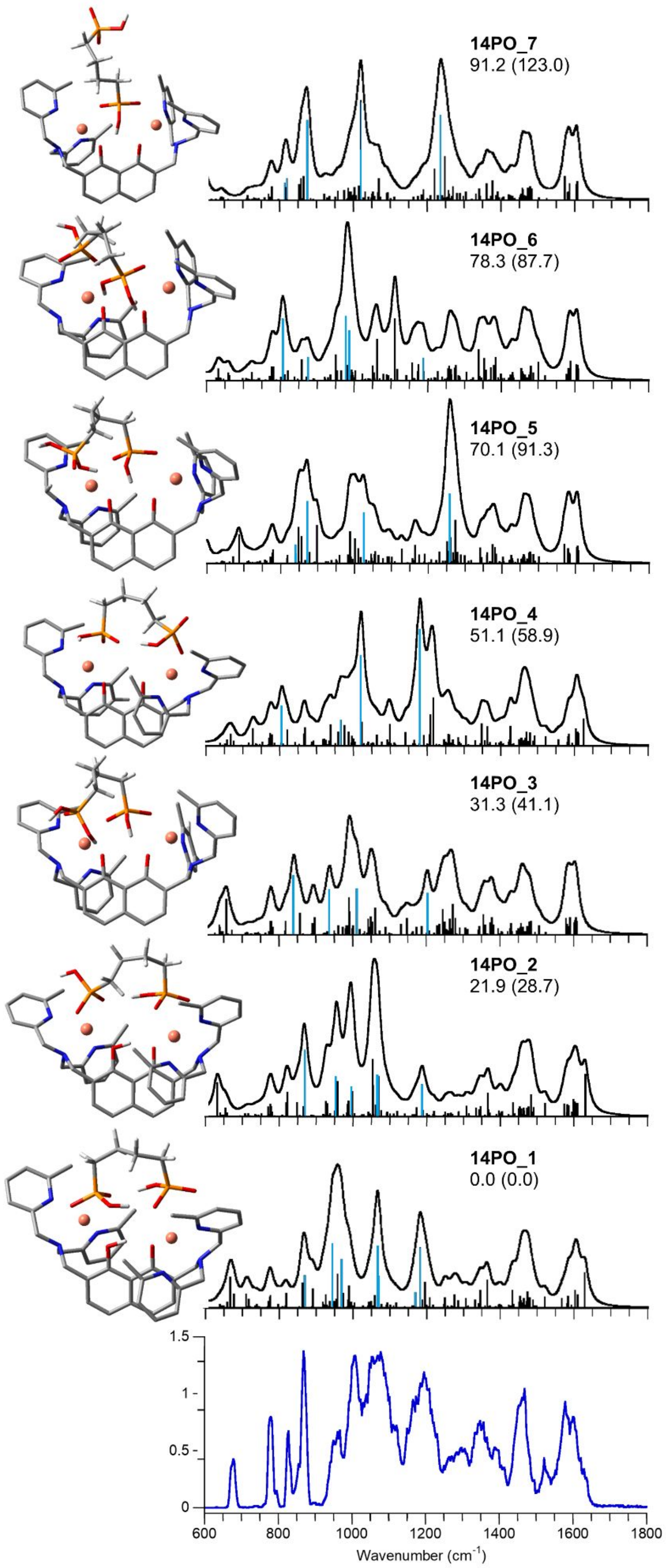

Figure S22. IRMPD spectrum of **[1(1,4PO)]**$^{+}$ (bottom panel) compared with calculated IR spectra of conformers and isomers, whose optimized structures are reported on the left. Relative free energies (enthalpies) at 298 K in kJ mol^{-1}. Unscaled vibrational modes are highlighted in pale blue.

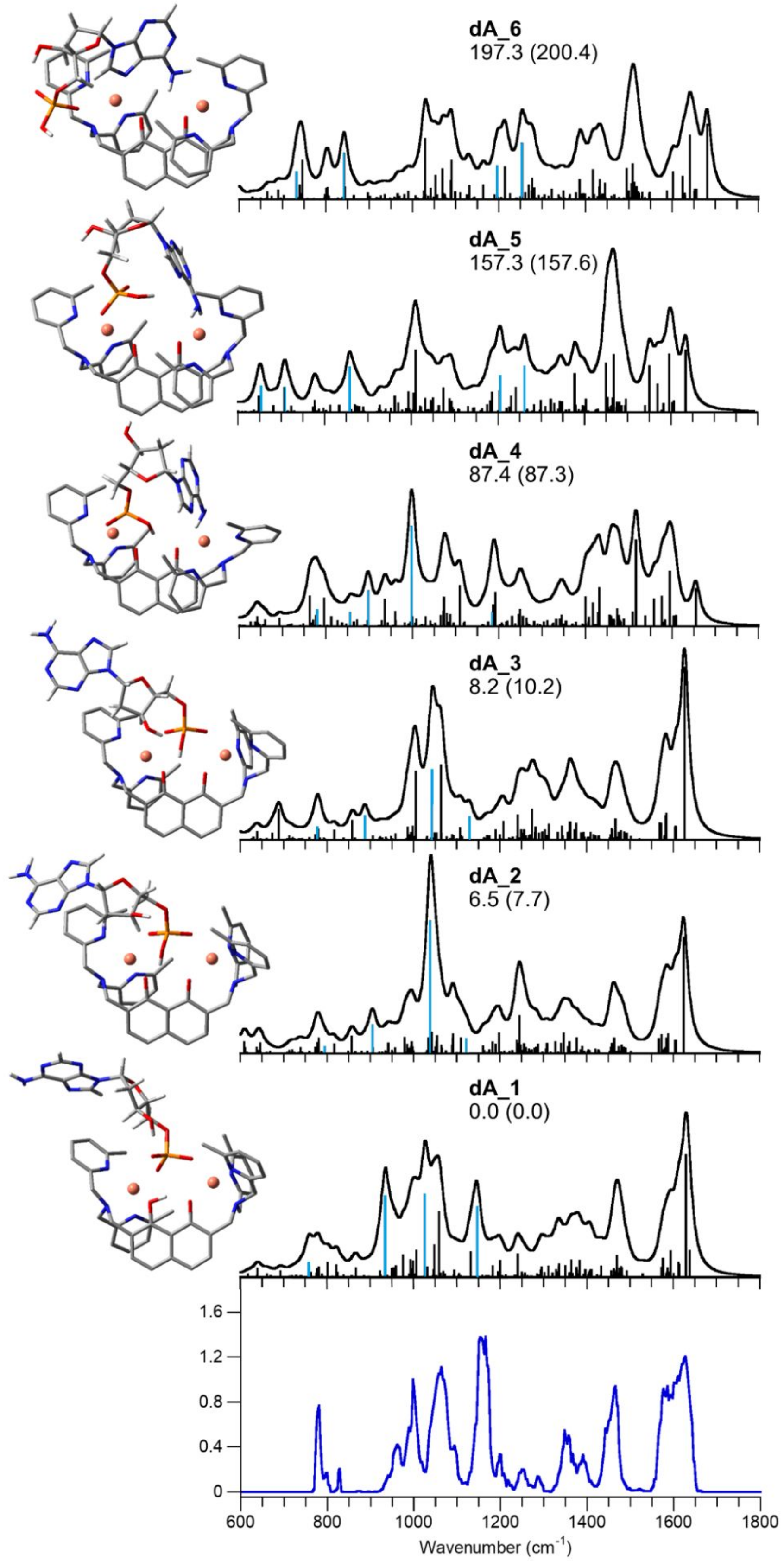

Figure S23. IRMPD spectrum of **[1(dAMP-2H)]**$^{+}$ (bottom panel) compared with calculated IR spectra of conformers and isomers, whose optimized structures are reported on the left. Relative free energies (enthalpies) at 298 K in kJ mol^{-1}. Unscaled vibrational modes are highlighted in pale blue.

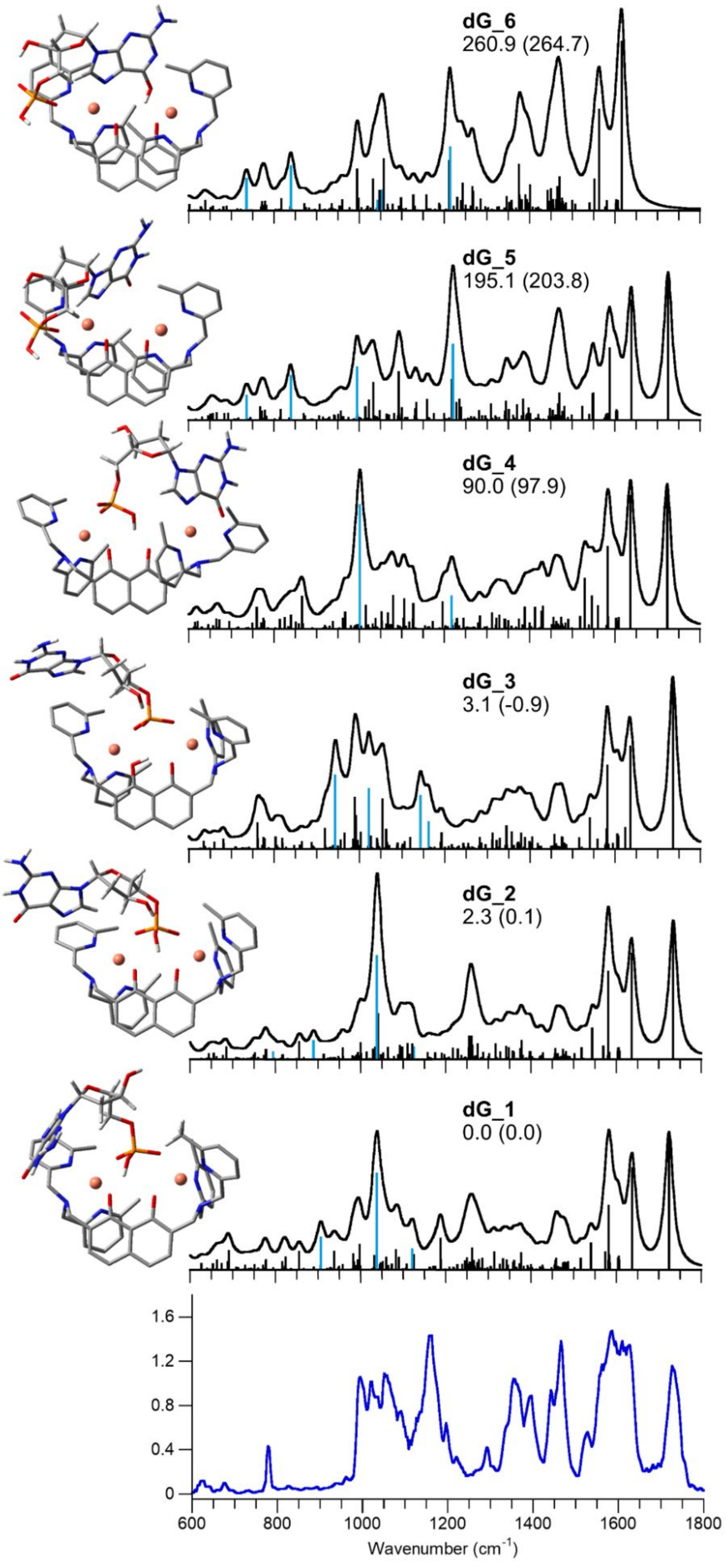

Figure S24. IRMPD spectrum of **[1(dGMP-2H)]**$^{+}$ (bottom panel) compared with calculated IR spectra of conformers and isomers, whose optimized structures are reported on the left. Relative free energies (enthalpies) at 298 K in kJ mol^{-1}. Unscaled vibrational modes are highlighted in pale blue.

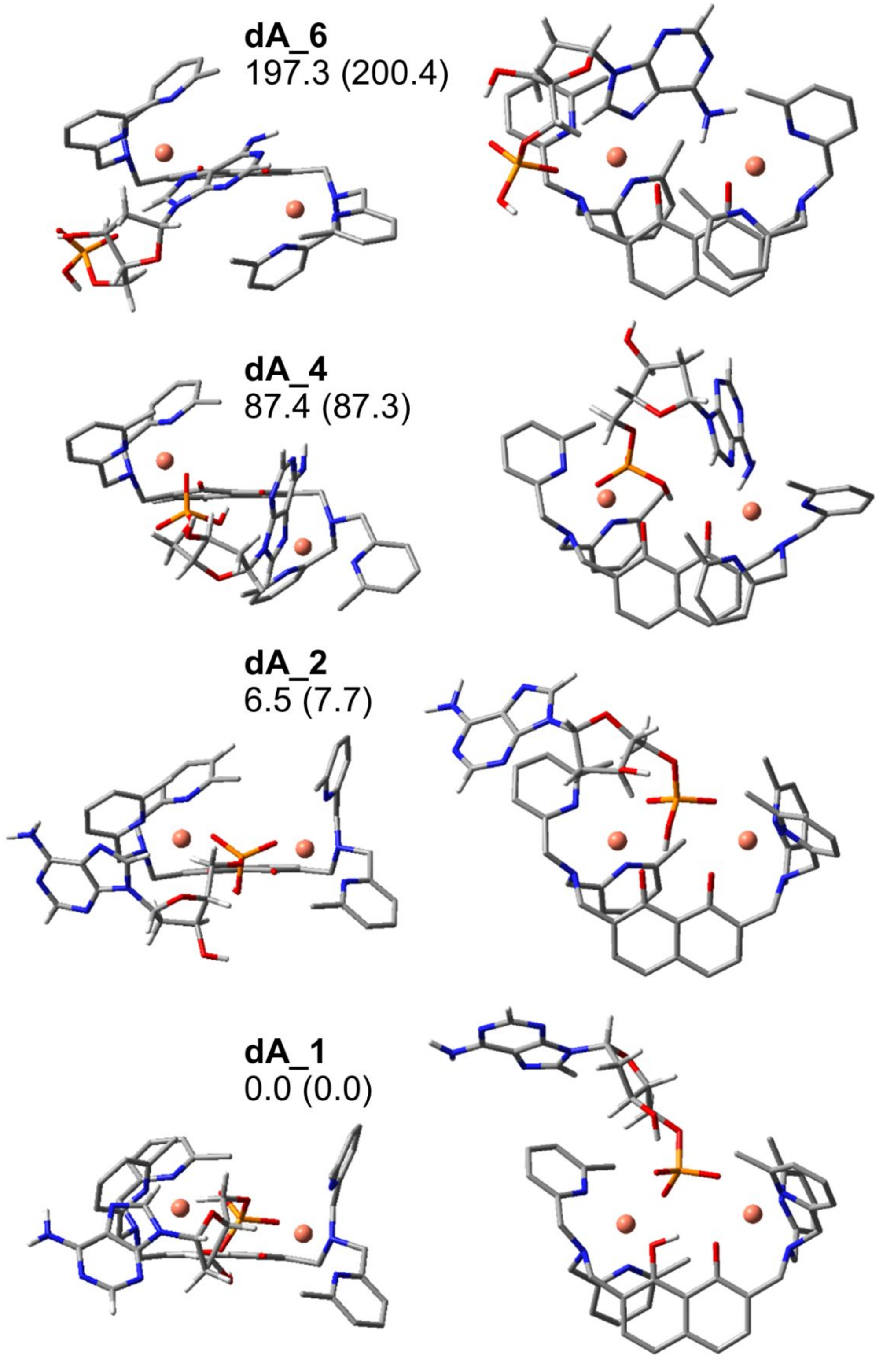

Figure S25. Optimized structures of **dA_1**, **dA_2**, **dA_4** and **dA_6** each from two different perspectives.

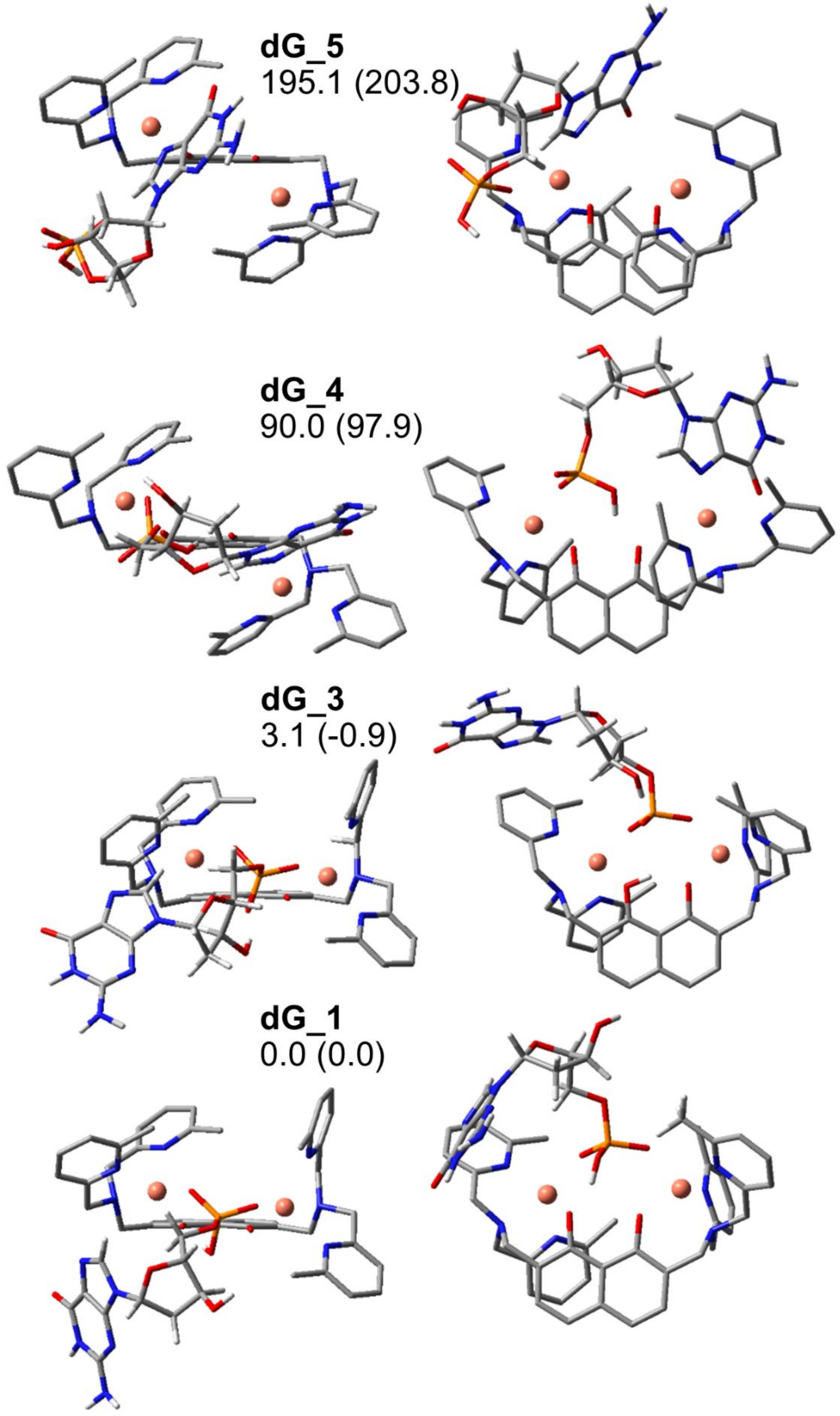

Figure S26. Optimized structures of **dG_1**, **dG_2**, **dG_4** and **dG_5** each from two different perspectives.

Table S1. Accurate (m_{acc}) and exact mass (m_{ex}) values of the sampled species obtained by high-resolution ESI(+) FT-ICR MS experiments from the reaction of [**1(OAc)$_2$**]$^+$ with phosphate ligands.

**Species** [a]	**Molecular Formula**	**m$_{acc}$**	**m$_{ex}$**	**Δppm**[b]
[**1(OAc)$_2$**]$^+$	$C_{44}H_{47}N_6O_6{}^{63}Cu_2$	881.21459	881.21436	0.3
[**1(HPO$_4$)**]$^+$	$C_{40}H_{42}N_6O_6P^{63}Cu_2$	859.14978	859.14900	0.9
[**1(1,2PO)**]$^+$	$C_{42}H_{47}N_6O_8P_2{}^{63}Cu_2$	951.15241	951.15171	0.7
[**1(1,4PO)**]$^+$	$C_{44}H_{51}N_6O_8P_2{}^{63}Cu_2$	979.18249	979.18301	-0.5
[**1(dAMP-2H)**]$^+$	$C_{50}H_{53}N_{11}O_8P^{63}Cu_2$	1092.24018	1092.24027	-0.1
[**1(dGMP-2H)**]$^+$	$C_{50}H_{53}N_{11}O_9P^{63}Cu_2$	1108.23550	1108.23519	0.3

[a] All species were revealed as singly charged ions.
[b] Error in parts per million (Δppm).

Table S2. Phenomenological threshold energies for the dissociation channels -L and -$C_{14}H_{17}N_3$ of [1(OAc)$_2$]$^+$, [1(HPO$_4$)]$^+$, [1(1,2PO)]$^+$, [1(1,4PO)]$^+$, [1(dAMP-2H)]$^+$, and [1(dGMP-2H)]$^+$ ions.

species	phenomenological threshold energy (eV)	
	-L[a]	-$C_{14}H_{17}N_3$[b]
[**1(OAc)$_2$**]$^+$ (m/z 881)	0.44 ± 0.2	nd[c]
[**1(HPO$_4$)**]$^+$ (m/z 859)	0.42 ± 0.2	0.70± 0.2
[**1(1,2PO)**]$^+$ (m/z 951)	nd[c]	0.53± 0.2
[**1(1,4PO)**]$^+$ (m/z 979)	nd[c]	0.55± 0.2
[**1(dAMP-2H)**]$^+$ (m/z 1092)	nd[c]	0.83± 0.2
[**1(dGMP-2H)**]$^+$ (m/z 1108)	nd[c]	0.83±0.2

[a] L = CH_3COOH, H_3PO_4, 1,2-POH_2, 1,4-POH_2, dAMP, and dGMP, respectively.
[b] $C_{14}H_{17}N_3$ corresponds to the neutral fragment bis-methylpyridine imino tridentate ligand from the tom^{Me} skeleton.
[c] Fragmentation path not detected in the inspected energy range (0 – 2.5 eV).

Table S3. Thermodynamic data for the optimized structures of **[1(OAc)$_2$]$^+$**, **[1(HPO$_4$)]$^+$**, **[1(1,2PO)]$^+$**, **[1(1,4PO)]$^+$**, **[1(dAMP-2H)]$^+$** and **[1(dGMP-2H)]$^+$**.

	B3LYP		*B3LYP-D3*		*M062X-D3*	
	H_{rel}	G_{rel}	H_{rel}	G_{rel}	H_{rel}	G_{rel}
**CuAc2_1**	0.0	0.0	0.0	0.0	0.0	0.0
**CuAc2_2**	-9.3	-2.9	-19.9	-13.5	-3.2	3.3
**CuAc2_3**	-2.9	1.5	-14.2	-9.8	1.9	6.3
**CuAc2_4**	25.7	21.1	28.8	24.2	40.8	36.2
**PO4_1**	0.0	0.0	0.0	0.0	0.0	0.0
**PO4_2**	27.6	34.2	24.8	31.5	14.6	21.2
**PO4_3**	37.7	42.9	30.3	35.5	19.7	24.9
**PO4_4**	41.7	46.9	31.3	36.4	23.6	28.7
**PO4_5**	43.2	49.2	34.5	40.5	33.2	39.2
**PO4_6**	44.3	51.0	59.5	66.2	84.7	91.4
**12PO_1**	0.0	0.0	0.0	0.0	0.0	0.0
**12PO_2**	21.7	26.1	17.8	22.3	5.8	10.3
**12PO_3**	30.6	48.5	17.1	35.0	6.3	24.2
**12PO_4**	12.8	9.7	22.0	18.9	27.5	24.3
**12PO_5**	27.8	30.8	27.0	30.0	23.9	26.9
**12PO_6**	31.1	17.3	50.5	36.6	56.7	42.8
**12PO_7**	41.8	43.3	37.6	39.1	43.0	44.5
**14PO_1**	0.0	0.0	0.0	0.0	0.0	0.0
**14PO_2**	25.6	18.9	31.5	24.7	28.7	21.9
**14PO_3**	25.2	15.4	25.3	15.5	41.2	31.3
**14PO_4**	31.4	23.6	48.7	40.8	58.9	51.1
**14PO_5**	41.6	20.5	66.6	45.5	91.3	70.1
**14PO_6**	69.9	60.4	80.4	71.0	87.7	78.3
**14PO_7**	66.8	35.0	102.6	70.8	123.0	91.2
**dA_1**	0.0	0.0	0.0	0.0	0.0	0.0
**dA_2**	-6.6	-7.8	-5.8	-7.0	7.7	6.5
**dA_3**	-7.4	-9.3	-6.5	-8.4	10.2	8.3
**dA_4**	20.9	21.0	17.5	17.6	87.4	87.5
**dA_5**	96.7	96.4	84.5	84.2	157.6	157.3
**dA_6**	103.6	100.4	101.5	98.3	200.4	197.3
**dG_1**	0.0	0.0	0.0	0.0	0.0	0.0
**dG_2**	-12.9	-10.7	-7.2	-4.9	0.1	2.3
**dG_3**	8.8	12.8	11.5	15.5	-0.9	3.1
**dG_4**	25.1	17.2	44.4	36.4	98.0	90.0
**dG_5**	109.5	100.9	124.1	115.4	203.8	195.1
**dG_6**	125.2	121.5	132.3	128.5	264.7	260.9

[a] Thermodynamic corrections from B3LYP harmonic frequency calculations.

Table S4. IRMPD features and theoretical IR frequencies (cm^{-1}), infrared absorption intensities (km mol^{-1}) in parenthesis, of **CuAc2_1**, **CuAc2_2**, **CuAc2_3**, **CuAc2_4** and vibrational normal modes obtained by DFT calculations at the B3LYP level.

IRMPD	Calculated				Assignment
	CuAc2_1	CuAc2_2	CuAc2_3	CuAc2_4	
1580	1638 (230)	1643 (198)	1629 (178)	1633 (155)	OH bend
		1603 (230)			CO_2 asym stretch
	1595 (69)	1598 (94)			CC stretch (naph)
		1594 (160)	1595 (207)	1592 (154)	CO_2 asym stretch
	1587 (61)				OH bend + CC stretch (naph)
			1572 (91)		OH bend
				1564 (196)	CO_2 asym stretch
1520	1525 (129)	1526 (106)			OH bend + CC stretch (naph)
				1523 (41)	CC stretch (naph) + CH bend ip (naph)
	1505 (130)		1505 (125)		CO_2 asym stretch
		1504 (72)			OH bend + CH_3 bends
1447		1488 (57)	1480 (82)	1489 (48)	CH_3 bends
	1465 (213)		1467 (62)	1483 (53)	
	1449 (155)			1470 (47)	
	1427 (72)			1452 (48)	
		1463 (79)	1430 (93)		CH bends
1373		1386 (143)	1386 (136)	1394 (167)	CH_3 umbrella (ac)
		1382 (138)		1387 (177)	
	1409 (67)		1368 (90)		CH_2 wagg
	1375 (50)				CC stretch (naph)
1327		1336 (242)	1337 (177)	1339 (278)	CH_3 umbrella (ac) + CO stretch (ac)
	1347 (45)				CH_2 wagg
		1330 (378)			
1287	1285 (28)	1288 (41)	1289 (26)	1289 (37)	CH_2 twist
1197	1192 (20)	1189 (13)	1190 (35)	1189 (18)	ring breath
1160	1166 (6)	1166 (10)	1165 (7)	1166 (8)	CH bend (pyr)
1090	1092 (12)	1094 (25)	1094 (11)	1094 (22)	
1053	1057 (43)	1062 (34)	1060 (42)	1057 (30)	CH bend + CH_2 twist
		1034 (72)		1038 (38)	Rings breath
997	986 (59)	987 (56)			CH bend + CH_2 rock
			987 (34)	987 (39)	Cu-N(Py) stretch + CH_3 twist
963				971 (64)	OH bend oop
	954 (74)	963 (27)	960 (27)		CH_2 rock
823	814 (54)	817 (62)	815 (51)	818 (61)	CH bend (Naph)
777	778 (34)	773 (51)	780 (45)	779 (56)	CH bend oop (Py)
680	665 (104)	650 (48)	667 (76)	656 (113)	CO_2 symm bend

Table S5. IRMPD features and theoretical IR frequencies (cm^{-1}), infrared absorption intensities ($km\ mol^{-1}$) in parenthesis, of **PO4_1**, **PO4_2** and vibrational normal modes obtained by DFT calculations at the B3LYP level. Unscaled vibrational modes are highlighted in blue.

IRMPD	Calculated		Assignment
	PO4_1	PO4_2	
1600		1631 (112)	CO-H bend
	1607 (61)	1606 (66)	Py rings breath
1573		1586 (34)	
	1585 (64)		CC stretch naph
	1573 (81)	1567 (43)	
1527		1523 (16)	OH bend + CH bend ip
	1503 (10)		CC stretch naph
1463	1482 (48)	1468 (51)	CH_3 asymm bend
1450	1457 (43)	1460 (41)	
		1429 (46)	CH bend ip + OH bend
1390	1388 (23)	1400 (30)	CH_3 umbrella
1360	1376 (63)	1377 (30)	CH_2 wagg
	1361 (54)	1366 (57)	
	1342 (51)	1331 (51)	
1290	1290 (29)	1289 (42)	CH_2 twist
1197	1190 (38)	1190 (29)	Naph ring breath
1163	1218 (215)		PO-H bend (H bonded)
		1184 (218)	PO stretch + PO-H bend
1077	1099 (53)		PO-H bend
		1090 (31)	CH_2 twist + CH bend
1050	1051 (133)		PO stretch
		1041 (214)	PO-H bend
1007	1001 (187)	1010 (317)	PO-H bend + PO stretch
963	958 (32)	954 (42)	CH_2 rock
	927 (153)	932 (273)	P-OH asym stretch
853	859 (72)	863 (31)	CC stretches (several)
827	818 (74)	818 (55)	CH bend oop (Naph)
777	778 (45)		CH bend oop (Py)
		784 (152)	P-OH stretch
		779 (39)	CH bend oop (Py)
